# In Situ Stress‐Dispersing Hydrogel Millispheres via Load Redistribution to Restore Nucleus Pulposus Metabolic Homeostasis

**DOI:** 10.1002/advs.75249

**Published:** 2026-04-13

**Authors:** Ang Li, Hui Yuan, Honglei Xiao, Xiaodong Liu, Wenguo Cui

**Affiliations:** ^1^ Department of Orthopedics School of Medicine Yangpu Hospital Tongji University Shanghai P. R. China; ^2^ Center For Clinical Research and Translational Medicine School of Medicine Yangpu Hospital Tongji University Shanghai P. R. China; ^3^ Department of Orthopaedics Shanghai Key Laboratory for Prevention and Treatment of Bone and Joint Diseases Shanghai Institute of Traumatology and Orthopaedics Ruijin Hospital Shanghai Jiao Tong University School of Medicine Shanghai P. R. China

**Keywords:** hydrogel millimeter spheres, intervertebral disc degeneration, metabolic homeostasis, stress dispersion

## Abstract

Abnormal mechanical stress can disrupt the metab homeostasis of the nucleus pulposus (NP) and induce inflammatory reactions and matrix degradation, thereby driving the progression of intervertebral disc degeneration (IDD). Owing to their small particle size, traditional micron hydrogel microspheres exhibit insufficient load dispersion, and it is difficult to effectively restore the stable environment of NP metabolism destroyed by local stress concentration. This study developed a bionic hydrogel millimeter sphere, approximately 1 mm in diameter, using a dual network design (HAMA/ChSMA, HA:ChS = 1:4) (ChS@HM) to realize the synergistic effect of mechanical dispersion and long‐term hydration lubrication. Finite element analysis showed that millimeter spheres can reduce the maximum stress on the spherical surface by approximately 50% compared to microspheres and reduce the stress concentration at the bottom by nearly 86%. In vitro experiments showed that continuous ChS@HM release of ChS alleviated the inflammatory microenvironment. Animal experiments have verified that it can promote matrix reconstruction. RNA sequencing revealed that ChS@HM regulates the Hippo/YAP signaling pathway, alleviates the inflammatory response and cell apoptosis induced by mechanical stress. This study innovatively developed an HA/ChS hydrogel millimeter sphere, which provides a new treatment strategy for IDD through an in situ stress dispersion mechanism.

## Graphical abstract

Local stress concentration disrupts metabolic homeostasis and induces inflammation in the nucleus pulposus (NP), thereby accelerating intervertebral disc degeneration (IDD). A biomimetic HA/ChS hydrogel millimeter sphere (ChS@HM) is developed to enable synergistic stress dispersion and sustained hydration lubrication. Modulating the Hippo/YAP pathway, it alleviates inflammation and apoptosis, facilitating matrix reconstruction in degenerated discs.

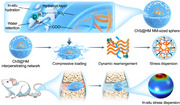

## 1 Introduction

The intervertebral disc (IVD) serves as a cushioning structure for the spine, allowing it to withstand complex stresses in multiple directions during daily activities [1, 2]. The nucleus pulposus (NP) relies on a high‐density hydration network centered on Aggrecan to achieve stress dispersion, where the glycosaminoglycan side chains (mainly ChS) on Aggrecan molecules adsorb a large number of water molecules through electrostatic interactions [3, 4] and interact with hyaluronic acid (HA) to form stable aggregates, jointly constructing a continuous and dense hydration structure [5]. This structure converts axial loads into uniformly distributed circumferential stresses through the expansion pressure formed by the high hydration state [6, 7]. In addition, NP further disperses stress under shear load through a dynamic hydration lubrication mechanism. At the macro level, hydration forms a fluid lubrication film under shear loads, thereby reducing interfacial friction and shear stress [8]; at the microscopic level, HA promotes the mutual sliding of collagen bundles in the IVD, preventing collagen damage under extreme loads [9]. Lubricants can reduce the torsional modulus and alleviate the damage caused by stress concentration to the IVD matrix [10]. However, with the degeneration of the IVD, the synthesis of Aggrecan and HA decreases, leading to a decrease in the hydration ability of NP [11]. Subsequently, the stress dispersion function of NP is gradually lost, leading to local stress concentration in the IVD, which in turn causes tissue structure damage and metabolic disorders of the cartilage matrix [12]. Therefore, reconstructing the hydration network of NP and restoring its stress dispersion ability are the key biomechanical targets for reversing IVD degeneration (IDD).

Some studies have attempted to restore the hydration network by injecting stable cross‐linked block hydrogel into NP [13]. However, its shape is irregular and prone to stress concentration at the edges. This research group has constructed a variety of hydrogel microspheres with a diameter of approximately 200 microns in the early stage and has achieved preliminary results in promoting the regeneration of NP [14, 15, 16]. However, small‐sized microspheres form a large number of contact points within the NP, resulting in irregular load paths and difficulty in achieving uniform stress dispersion. Meanwhile, frequent relative motion and multiple interfaces between microspheres may disrupt the continuity of the hydration lubrication film and weaken the lubrication efficiency. According to the classical Hertz contact theory, increasing the radius of the sphere can expand the contact area and reduce the unit contact pressure under small deformation conditions [17]. Recent studies have shown that under high compression ratio conditions, a single elastic sphere can still maintain a large contact area and low unit contact pressure, thereby forming a stable force interface [18]. Although the above theory does not directly involve a multi‐sphere system, it can be inferred from the multi‐sphere contact simulation results that the use of a larger single hydrogel sphere will help reduce the number of interfaces, simplify the load path, and maintain a continuous structure of the hydration lubrication film, thus improving the overall stress dispersion performance.

The effectiveness of the stress dispersion strategy depends not only on the mechanical properties of the material itself but also on the pathological microenvironment of degenerated NP. As IDD progresses, concentrated stress promotes NP inflammation via NLRP3 [19]. Moreover, the upregulation of pro‐inflammatory factors TNF‐α and IL‐1β significantly induces the overexpression of matrix metalloproteinases (MMPs) and accelerates the degradation of Aggrecan and HA [20]. This process disrupts the hydration network of NP, exacerbates local stress concentration, and aggravates degeneration. Chondroitin sulfate (ChS), as the most abundant glycosaminoglycan in NP, can maintain tissue swelling pressure through high hydration and has certain anti‐inflammatory activity [21]. However, the use of ChS alone has limitations in terms of mechanical properties and rapid degradation [22], and a combination of ChS and HA can enhance the viscoelasticity and stability of the entire system [23]. Therefore, constructing an in situ stress dispersion platform based on HA/ChS is expected to restore the hydration lubrication network of NP, regulate the inflammatory response, and inhibit the progression of IDD.

**SCHEME 1:**
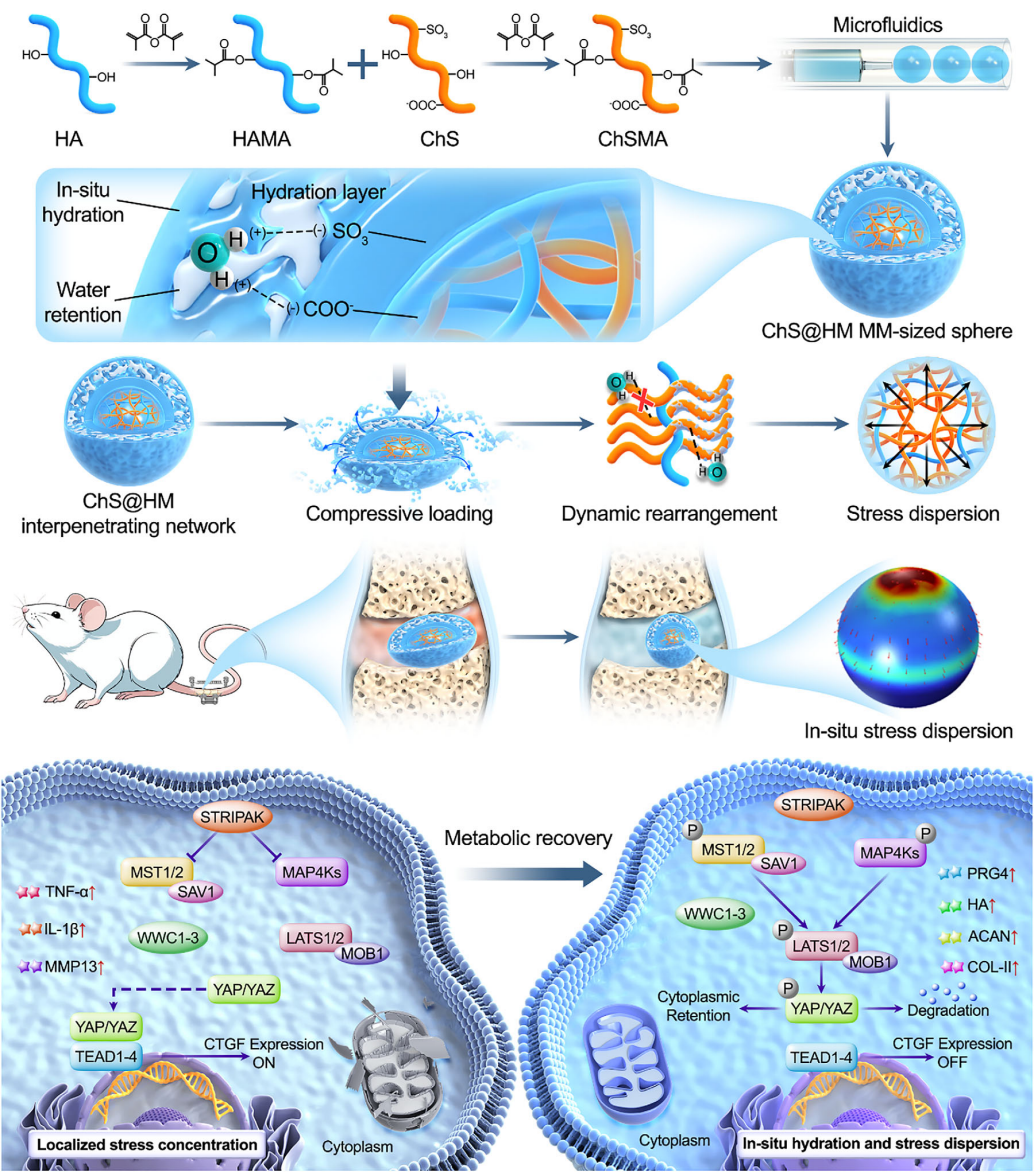
Schematic design and experimental flow diagram.

In this study, a biomimetic hydrogel millimeter sphere with a diameter of 1 mm was innovatively constructed by combining microfluidic and photocross‐linking technologies. Specifically, according to the composition [24] and size [25] of natural NP, methacryloylated hyaluronic acid (HAMA) and methacryloylated chondroitin sulfate (ChSMA) were mixed to prepare stable ChSMA/HAMA hydrogel millimeter spheres (ChS@HM). At the macroscopic scale, an in situ stress dispersion network was constructed through cross‐linked spheres, while at the microscopic level, a continuous and dense hydrated lubricating film was retained. Moreover, in vitro tribological characterization and finite element simulation were performed. The long‐lasting hydration, lubrication performance, and significant stress dispersion characteristics of the product were further confirmed by cell model experiments, which confirmed its sustained release of ChS and inhibitory effect on inflammatory cytokines in NP cells. In a rat model of caudal vertebral compression degeneration, its promoting effect on IVD height, water content, and matrix protein reconstruction was validated through imaging and histological analysis. The strategy of integrating in situ stress dispersion and hydration lubrication provides a new solution for IDD repair that combines mechanical and biological biomimetic approaches (Scheme 1).

## 2 Results and Discussion

### 2.1 Preparation and Characterization of Hydrated Lubrication Hydrogel Millimeter Spheres

**FIGURE 1:**
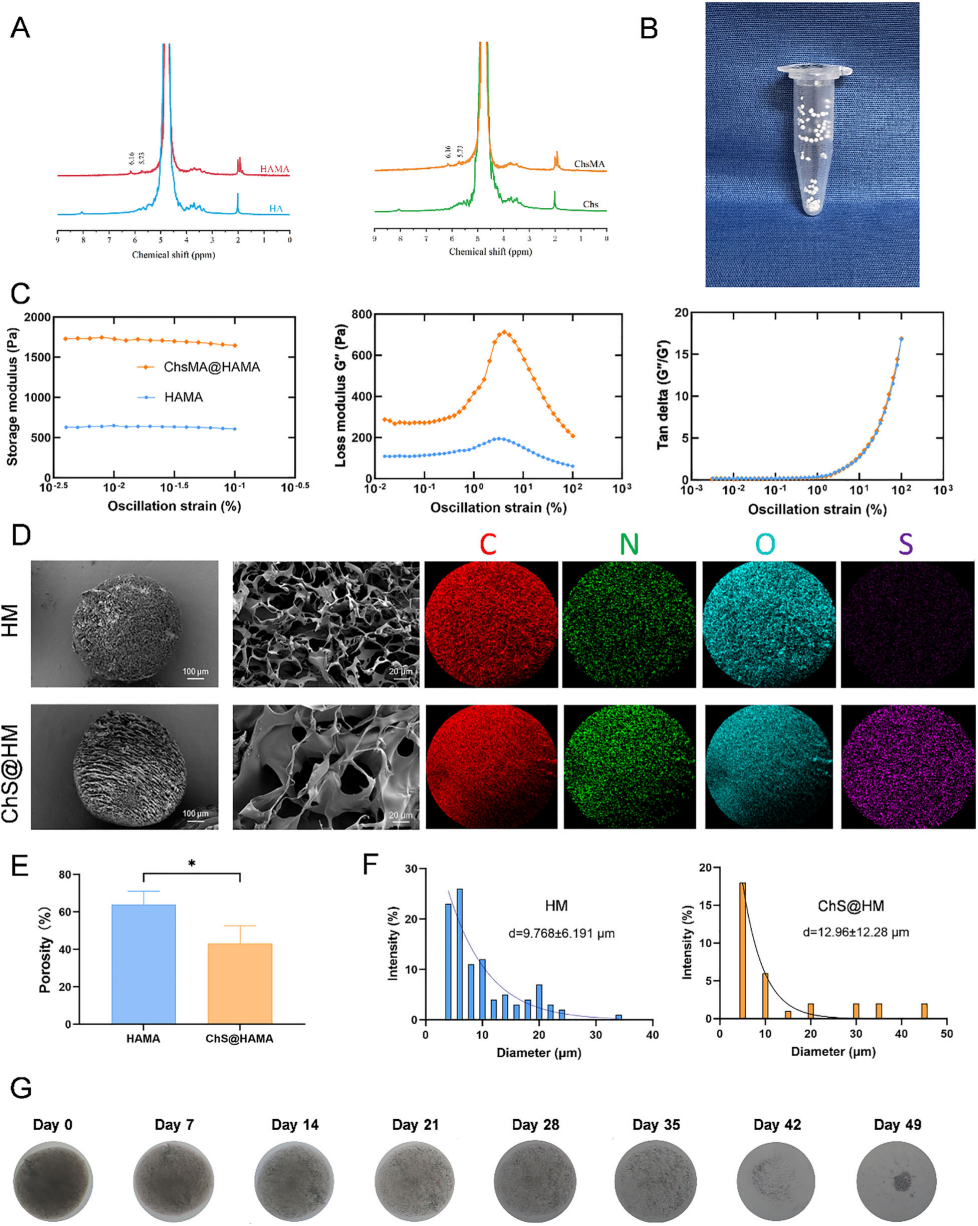
Characterization of Hydrogel Millimeter Spheres. (A) ^1^H NMR spectra of HAMA and ChSMA. (B) Macroscopic image of ChS@HM millimeter spheres after freeze‐drying. (C) Rheological testing of HAMA and ChS@HM. D) SEM images and element distribution images of HAMA and ChS@HM millimeter spheres. (E) Porosity of HAMA and ChS@HM millimeter spheres (*n* = 3). (F) Pore size of HAMA and ChS@HM millimeter spheres. (G) Degradation image of ChS@HM sphere in vitro. Quantitative data are presented as mean ± SD. Error bars represent SD. Statistical analysis was performed using an unpaired two‐tailed Student's *t*‐test. ^*^ *p* < 0.05.

In this study, methacrylic anhydride (MA) was first used to modify HA and ChS. The 1H NMR spectrum results (Figure 1A) show new characteristic peaks at δ = 6.16 and 5.73 ppm, indicating that MA was successfully grafted onto HA and ChS molecules. Quantitative calculation of the degree of substitution was performed based on the integral area ratio of the methacryloyl proton peaks to the N‐acetyl methyl proton peaks of HA or ChS. The results demonstrated that the methacrylation substitution degrees of HAMA and ChSMA were approximately 35.6% and 66.7%, respectively. Subsequently, microfluidic technology was used to prepare a HAMA/ChSMA dual network with an average diameter of 1 mm, based on a biomimetic NP ratio of HA:ChS = 1:4. Millimeter spheres have good macroscopic structural stability, providing a foundation for maintaining local spatial morphology and mechanical functions in vivo in the future (Figure 1B).

The rheological test results showed that the storage modulus (G') of the HM group remained at approximately 620‒650 Pa within a small strain range (0.004‒0.1), while that of ChS@HM significantly increased to 1650‒1750 Pa, which is approximately 2.5‒3 times higher, indicating that the dual network system has a more stable elastic skeleton. The corresponding loss modulus (G″) results show that the HM group remained at approximately 100‒150 Pa, while that of ChS@HM significantly increased to approximately 270‒700 Pa, indicating that it has a stronger energy dissipation ability under external forces, which helps to buffer cyclic loads. It is worth noting that the loss tangent (tan δ) curves almost overlap in the entire strain range, indicating that although the overall modulus of ChS@HM is higher, its elasticity‐to‐viscosity ratio remains consistent with that of HM, indicating that the material still exhibits elastic‐dominant characteristics (Figure 1C). Due to the simultaneous improvement in elasticity and dissipation capacity, the tanδ curve remains basically stable. This mechanical property is beneficial for providing a microenvironment for NP that is both stable and cushioning, helping to maintain tissue metabolism and function.

SEM observations showed that the HM and ChS@HM spheres exhibited obvious pore structures, and CNOS elemental analysis showed that the surfaces of the two groups of spheres were mainly composed of C, N, and O elements, while the presence of the S element was additionally detected in ChS@HM, confirming the successful introduction of ChS (Figure 1D). Further analysis revealed that ChS@HM porosity decreased compared to that of HM (43.13 ± 9.43 vs 64.09 ± 7.1%) (Figure 1E), while the average pore size slightly increased (12.96 ± 12.28 vs 9.768 ± 6.191 µm) (Figure 1F). Larger pore size and higher porosity facilitate hydration and molecular diffusion but may also lead to a decrease in the bulk mechanical behavior of the scaffold [26]. However, although an increase in aperture is usually accompanied by a decrease in mechanical stability, as the ChS@HM pore size increases, the porosity decreases, enhancing the continuity of the pore wall and balancing hydration and mechanical stability. Studies have found that pore size and porosity can synergistically regulate the water absorption and mechanical behavior of porous scaffolds. Especially in the hydrated state, the dependence of elastic modulus on pore structure decreases, while the energy dissipation capacity increases, facilitating uniform load transfer and local stress relief [27]. This suggests that the changes in the pore structure of ChS@HM may improve the microenvironment of NP cells through a similar mechanism. Atomic force microscopy revealed that the surface of ChS@HM exhibits a continuous nanoscale morphology with a surface roughness of Rq ≈ 307 nm and Ra ≈ 238 nm, further confirming the continuity of its structure at the nanoscale (Figure S1). In addition, the ChS@HM sphere exhibited good degradation characteristics. The in vitro degradation experiment simulated by hyaluronidase showed that ChS@HM was 86% degraded by day 49 (Figure 1G). These findings indicate that the ChS@HM sphere has a moderate pore size, porosity, and degradation rate that match tissue repair, which helps with hydration and stress dispersion and provides a structural and functional basis for the regulation of NP metabolism homeostasis.

The swelling behavior of HM and ChS@HM spheres was examined by tracking their mass change in PBS over time. HM spheres swelled rapidly and reached equilibrium within 1 h, with a swelling ratio of approximately 195 ± 9.85%. In comparison, ChS@HM spheres showed a slightly lower swelling ratio of about 164.33 ± 11.72%. Both groups remained stable at 4 h, indicating that equilibrium had been achieved. The reduced swelling of ChS@HM spheres is likely due to the denser network formed after ChS incorporation, which restricts initial water uptake. After full swelling, the spheres were allowed to dehydrate in air to evaluate their water retention capacity. Over time, HM spheres lost water more quickly, while ChS@HM spheres retained more water, indicating better water retention (Figure S2). This difference may be explained by the abundant sulfate and carboxyl groups on ChS, which form a mobile hydration layer with water and help maintain hydration during dehydration.

To investigate the release behavior of ChS from ChS@HM, an in vitro release assay was performed (Figure S3). The results showed a sustained release profile. An initial modest burst release was observed, with approximately 5.12% of ChS released within the first hour. This was followed by a gradual increase, reaching 22.67% at 24 h. Thereafter, the release rate slowed, with a cumulative release of 32.67% at 168 h, indicating that ChS can be continuously released from ChS@HM.

#### 2.1.1 Finite Element Analysis

**FIGURE 2:**
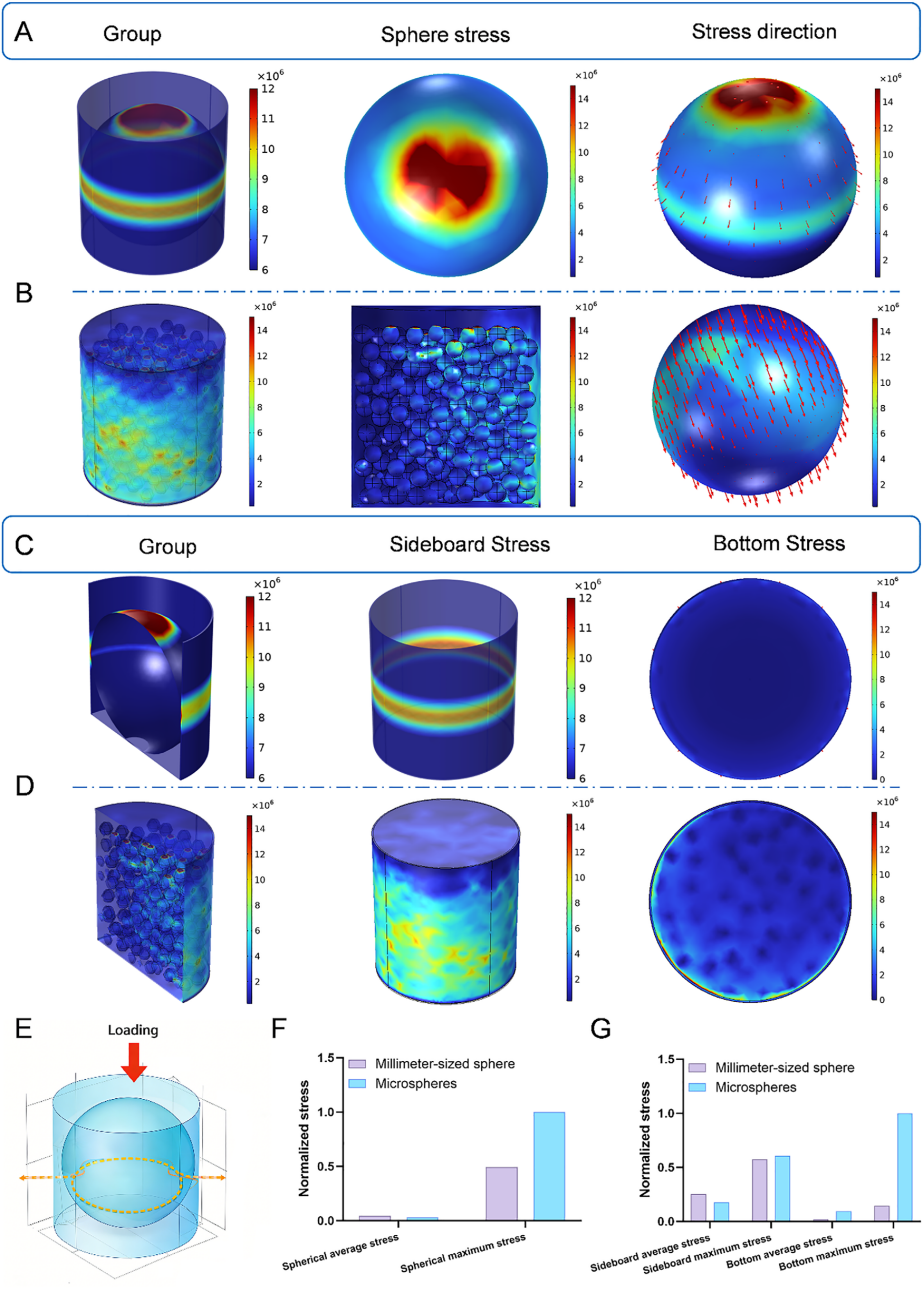
Finite element analysis of millimeter‐sized spheres and microspheres under top loading conditions. (A) Average stress and stress dispersion direction of a millimeter sphere. (B) Average stress and stress dispersion direction of microspheres. (C) Stress distribution of millimeter spheres on the container's side walls and bottom. (D) Stress distribution of microspheres on the container's side walls and bottom. (E) Schematic diagram of loading direction for finite element analysis of millimeter spheres. (F) Quantitative comparison of average spherical stress and maximum spherical stress between two sets of spheres. (G) Quantitative comparison of average sidewall stress, maximum sidewall stress, average bottom stress, and maximum bottom stress between two sets of spheres. Data in F and G are from single finite element simulations; error bars are not applicable.

To evaluate the stress dispersion performance of millimeter spheres and microspheres under stress, finite element analysis was conducted on both structures. The results show that the overall stress on the surface of the millimeter sphere was slightly higher, but the maximum stress was significantly reduced, indicating that it could more effectively disperse local stress during the compression process (Figure 2A,B,F). The stress distribution in the sidewall area was relatively uniform, indicating that the stress was transmitted more widely to the surrounding areas rather than being concentrated in a single area. Because of the increase in the stress diffusion range, the average stress in the local sidewall area slightly increases, which is a manifestation of the overall stress dispersion effect, especially in the bottom region, where the average and peak stresses of millimeter spheres are significantly lower than those of microspheres, indicating that they can significantly alleviate the stress concentration at the bottom (Figure 2C,D,G). This simulation used a normalized stress representation to compare the stress distribution trends of different structures. Overall, millimeter spheres have the potential to exhibit excellent stress dispersion ability in NP, which can more evenly transmit external forces to the surrounding matrix, thereby reducing the risk of local damage.

#### 2.1.2 Friction Test

**FIGURE 3:**
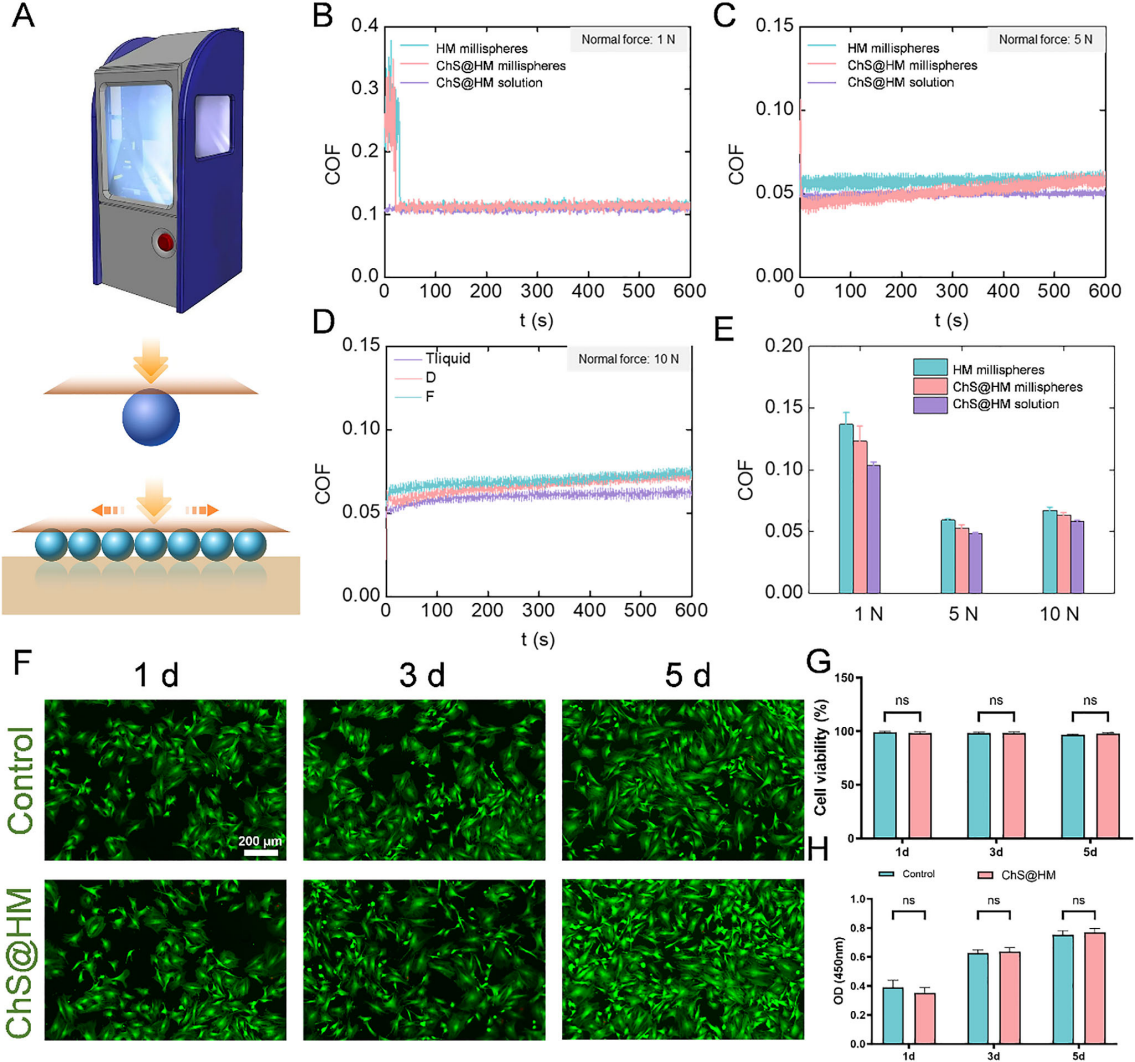
Friction testing and biocompatibility testing. (A) Photos and friction test schematic of SRV5. (B) Time curve of ChS@HM at a normal force of 1 N (*n* = 3). (C) Time curve of ChS@HM at a normal force of 5N (*n* = 3). (D) Time curve of ChS@HM at a normal force of 10 N (*n* = 3). (E) Corresponding average COF of ChS@HM (*n* = 3). (F, G) Live and dead ChS@HM staining images of cocultured rat NP cells (*n* = 3). (H) CCK8 assay used to evaluate cell viability (*n* = 3). Results are expressed as the average value with corresponding standard deviation, and variability is indicated by standard deviation in the graphs. Statistical analysis was performed using an unpaired two‐tailed Student's *t*‐test for comparisons between two groups, and one‐way ANOVA followed by Tukey's multiple comparisons test for multiple group comparisons. ns *p* > 0.05.

To evaluate the lubrication performance of different materials, different loads were set at a shear frequency of 1 Hz for the HM and ChS@HM millimeter spheres, and the solution underwent linear friction testing (Figure 3A). In a non‐load bearing state, the range of pressure that the human lumbar IVD can withstand during daily activities is approximately 0.8‒3 times body weight [28], while the average body weight of 10‐week‐old SD rats is 350 g [29], corresponding to a force of 3.43 N being approximately 1 times their body weight. Based on this, this study established normal forces of 1 N, 5 N, and 10 N for lubrication testing, equivalent to 0.29‒2.9 times the body weight of rats, which can better simulate the physiological stress conditions of IVD. When the normal force is 1 N, the average COF values of the HM millimeter sphere, ChS@HM millimeter sphere, and ChS@HM solutions were 0.136 ± 0.014, 0.125 ± 0.012, and 0.107 ± 0.01, respectively, with the most significant differences (Figure 3B). Under the conditions of 5 and 10 N, the increased contact pressure may partially compress the hydration layer, leading to the differences among the three groups decreased, but ChS@HM millimeter spheres were still superior to HM millimeter spheres (Figure 3C,D). ChS exhibited a higher water content than hyaluronic acid because its sulfuric acid group can bind additional water molecules; however, these water molecules bound by sulfate groups have relatively weak hydrogen bonds, resulting in a looser hydration layer structure and higher fluidity [30]. This feature not only enhances the overall hydration of ChS@HM millimeter spheres but also provides favorable conditions for interface lubrication.

### 2.2 In Vitro Hydrated Lubricating Millimeter Spheres Promote Stable Metabolism of the Nucleus Pulposus

ChS maintains the high hydration state of NP [31] and effectively inhibits inflammatory reactions in NP [13]. Adopting ChS@HM to reduce the expression of inflammatory factors and restore ECM synthesis and NP catabolism balance is a key strategy for IDD treatment.

**FIGURE 4:**
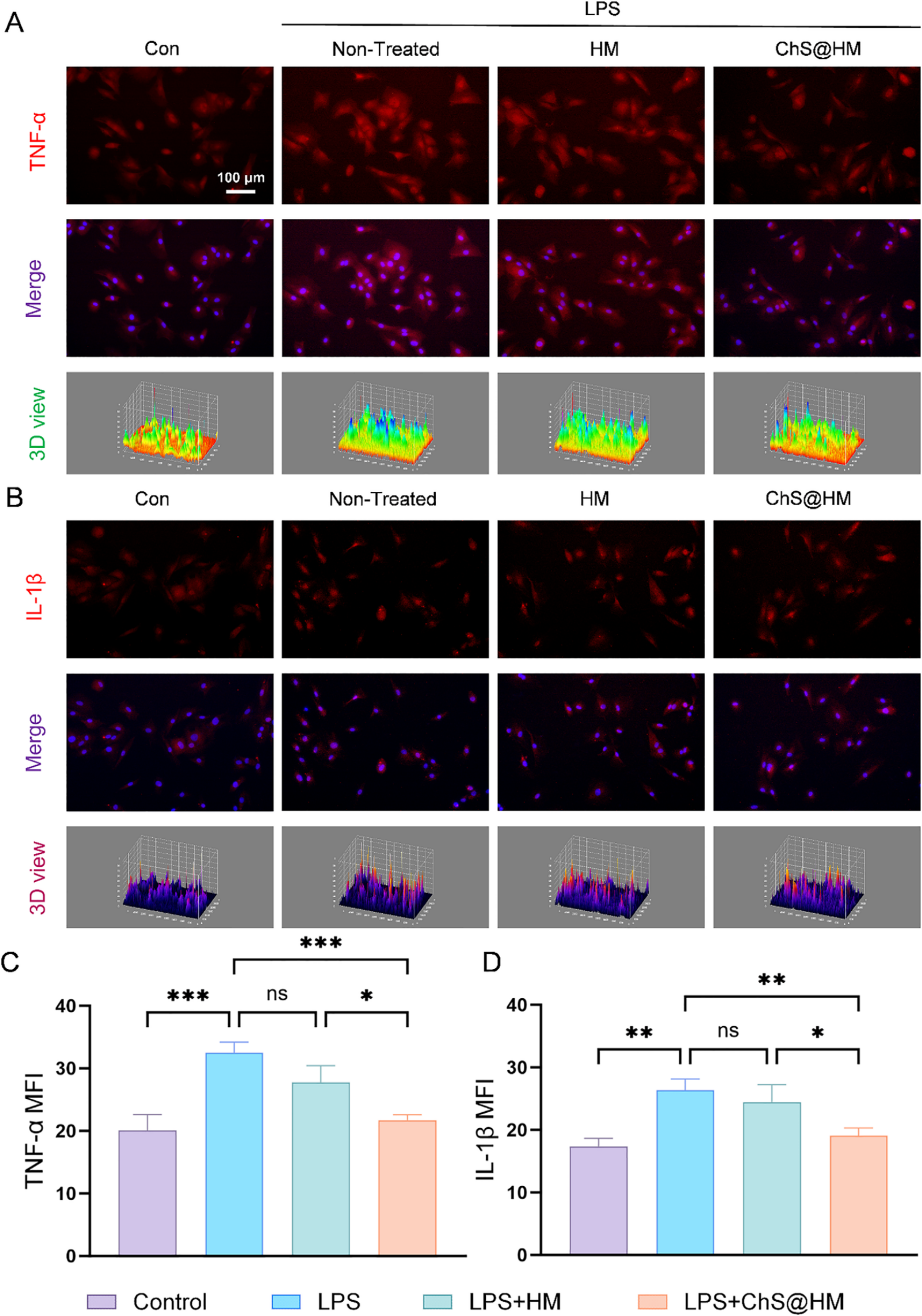
Expression analysis of inflammatory markers in NP cells cultured in LPS for 12 h. (A) Immunofluorescence staining of TNF‐α. (B) Immunofluorescence staining of IL‐1β. (C) Semi‐quantitative assessment of TNF‐α immunofluorescence intensity (*n* = 3). (D) Semi‐quantitative assessment of IL‐1β immunofluorescence intensity (*n* = 3). Results are expressed as the average value with corresponding standard deviation, and variability is indicated by standard deviation in the graphs. Group differences were analyzed using one‐way analysis of variance with subsequent Tukey post hoc testing. ns *p* > 0.05, ^*^ *p* < 0.05, ^**^ *p* < 0.01, ^***^ *p* < 0.001.

The therapeutic impact of inflammation inhibition in NP was assessed by analyzing ChS@HM through immunofluorescence staining, focusing on TNF‐α and IL‐1β expression levels post‐co‐culture in each group. Following a 12‐h LPS treatment of rat NP cells, TNF‐α and IL‐1β expression levels rose in all treatment groups relative to the Control group, with ChS@HM exhibiting the lowest expression among them (Figure 4A–D). The qPCR and ELISA results also support this result (Figures S4 and S5).

**FIGURE 5:**
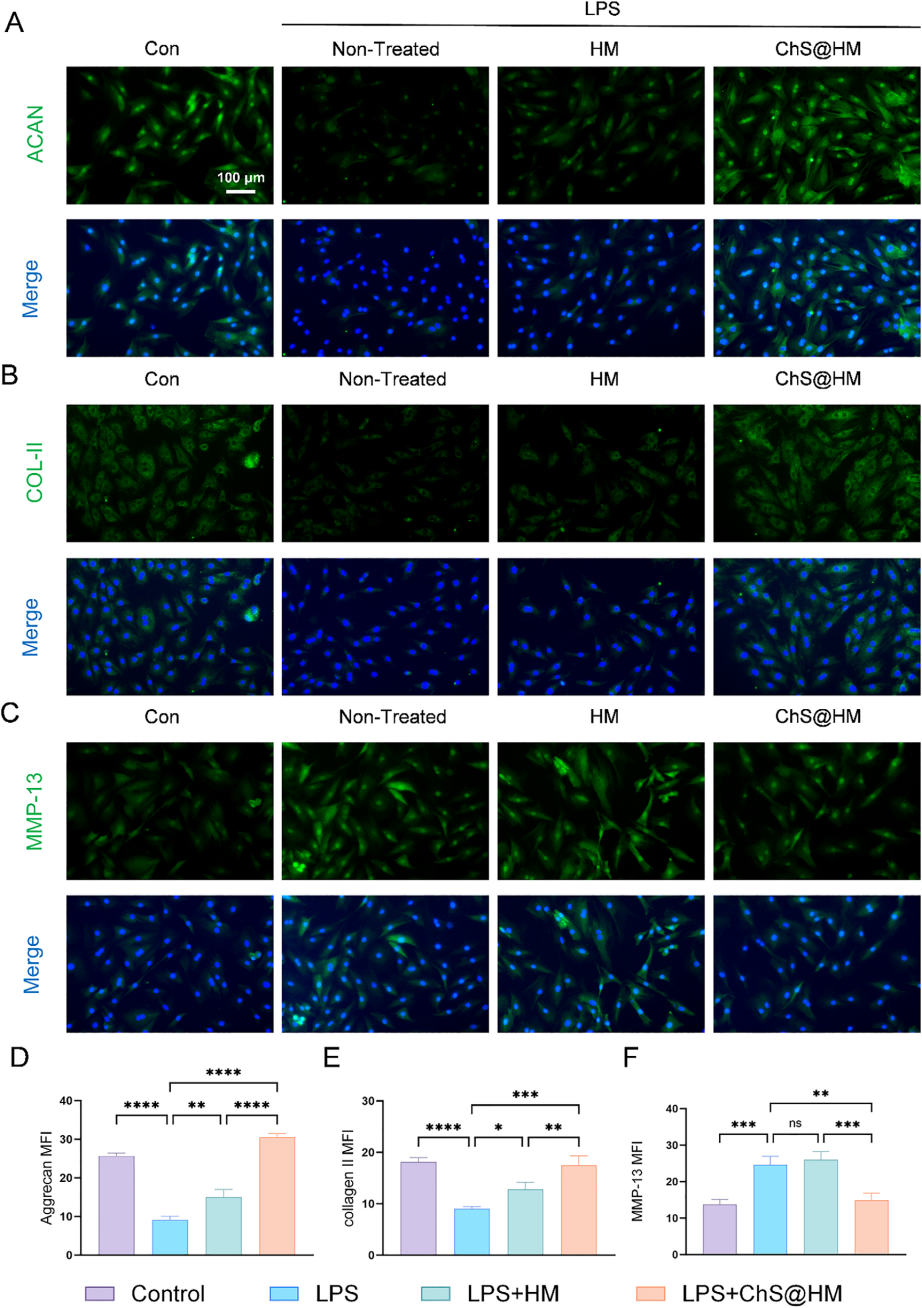
Analysis of synthetic catabolism markers in NP cells exposed to LPS for 12 h. (A) Aggrecan immunofluorescence staining. (B) COL‐II immunofluorescence staining. (C) MMP‐13 immunofluorescence staining. (D) Semi‐quantitative assessment of Aggrecan immunofluorescence intensity (*n* = 3). (E) Semi‐quantitative assessment of collagen‐II immunofluorescence intensity (*n* = 3). (F) Semi‐quantitative assessment of MMP‐13 immunofluorescence intensity (*n* = 3). Results are expressed as the average value with corresponding standard deviation, and variability is indicated by standard deviation in the graphs. Group differences were analyzed using one‐way analysis of variance with subsequent Tukey post hoc testing. ns *p* > 0.05, ^*^ *p* < 0.05, ^**^ *p* < 0.01, ^***^ *p* < 0.001, ^****^ *p* < 0.0001.

Furthermore, considering that the process of ECM reconstruction involves the breakdown and synthesis of ECM components, we investigated the expression levels of NP cell synthesis catabolic proteins, including Aggrecan, COL‐II, and MMP‐13, using immunofluorescence technology. Among the four treatment groups, ChS@HM maintained the highest Aggrecan (Figure 5A,D) and COL‐II (Figure 5B,E), as well as the lowest expression level of MMP‐13 (Figure 5C,F). The qPCR results also support this result (Figure S6). In vitro experiments showed that ChS@HM can effectively inhibit inflammation while rebuilding the ECM of NP cells.

### 2.3 In Vivo Hydrated Lubricating Millimeter Spheres Promote Stable Metabolism of the Nucleus Pulposus

**FIGURE 6:**
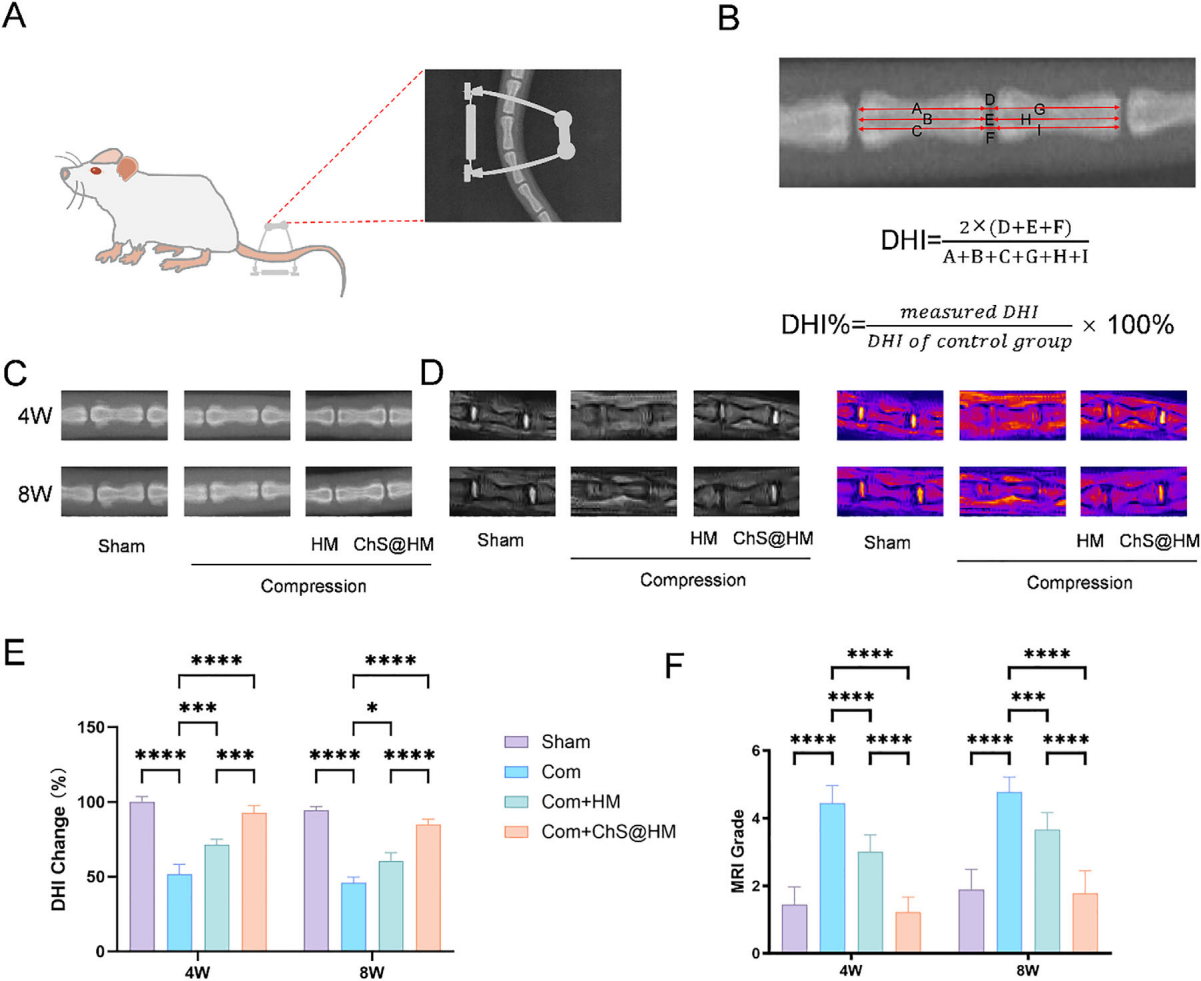
Imaging evaluation of rat IVD degeneration model. (A) Schematic diagram of rat caudal vertebral compression model. (B) Schematic diagram for calculating changes in IVD height. (C) Postoperative X‐ray images of IVD at two different time points. (D) Postoperative MRI images and IVD heatmaps at two different time points. (E) Postoperative changes in DHI at 4 and 8 weeks during IVD (*n* = 6). (F) Postoperative MRI grading alterations at two different time points (*n* = 6). Results are expressed as the average value with corresponding standard deviation, and variability is indicated by standard deviation in the graphs. Group differences were analyzed using one‐way analysis of variance with subsequent Tukey post hoc testing. ^*^ *p* < 0.05, ^***^ *p* < 0.001, ^****^ *p* < 0.0001.

ChS@HM millimeter spheres can capture free water to form a persistent hydrated lubricating layer. To further examine the therapeutic impact of millimeter spheres on NP regeneration in vivo, a static compression IDD model was established in rats using a self‐made compression device, and radiological evaluations were recorded at 4 weeks and 8 weeks after surgery (Figure 6A). The intervertebral disc height index (DHI) was observed from X‐ray images of the rat tail, which reflected changes in the IVD space (Figure 6B). Rats were treated with HM and ChS@HM millimeter spheres. The X‐ray images at two different time points after surgery showed a statistically significant reduction in DHI in the compression group relative to the sham group. The ChS@HM group maintained DHI levels to the greatest extent possible, while the other two treatment groups maintained the collapse of IVD space (Figure 6C,E). According to the improved Thomson grading method, MRI signal intensities were categorized into grades I to IV. A weak signal strength indicates low moisture content, and a high level represents severe degradation. The grading of the three compressed groups of IVD increased after 4 and 8 weeks, and it was observed that ChS@HM maintained the grading of IVD to the greatest extent possible (Figure 6D,F). These results indicate that ChS@HM can maintain the structural integrity of IVD.

**FIGURE 7:**
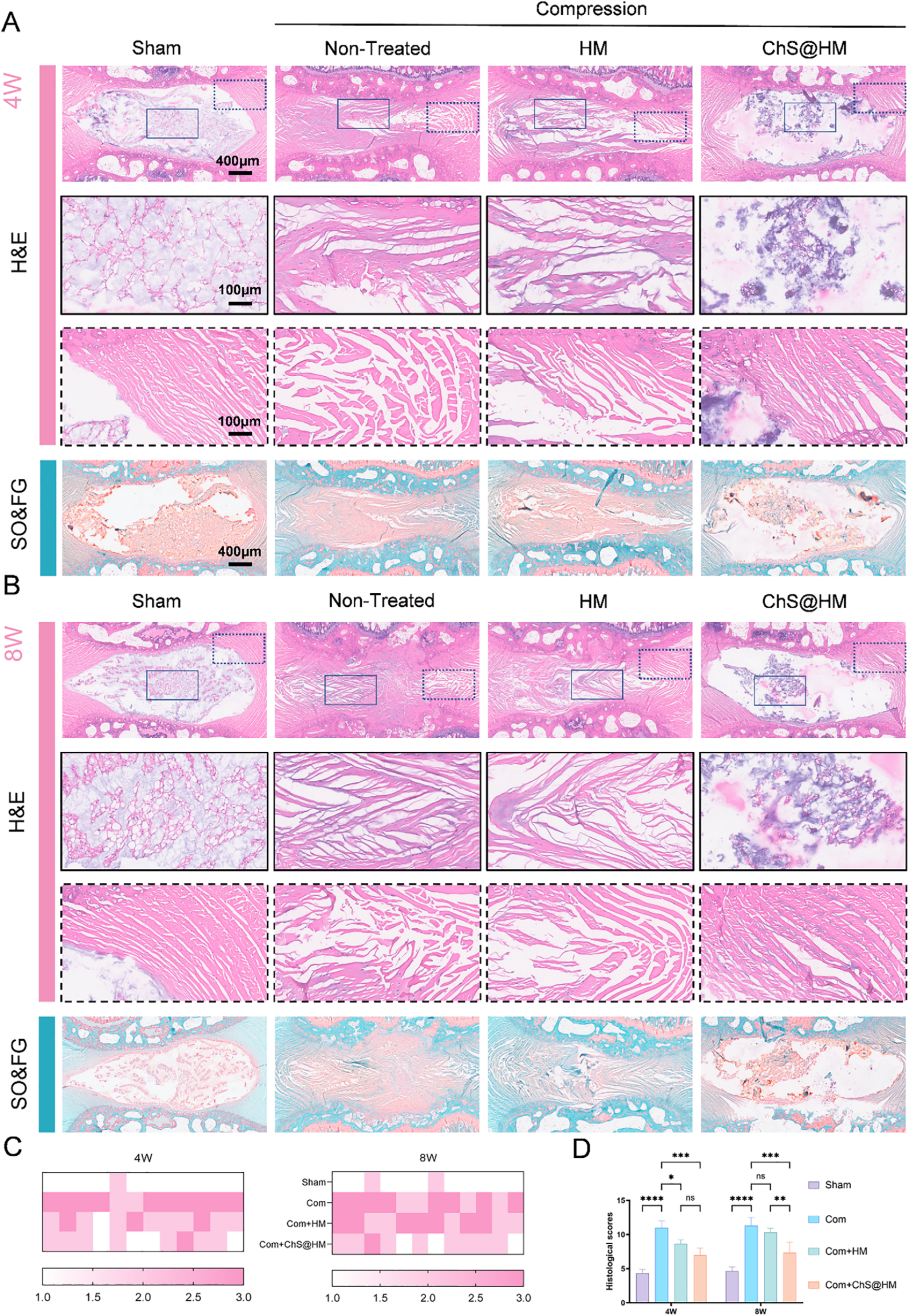
Histological evaluation of animal studies. (A, B) Postoperative H&E and safranin green stained images of IVDs at 4 and 8 weeks. (C) Heatmap illustrating histological grading of IVDs at 4 and 8 weeks after surgery. (D) Histological evaluation of IVDs at 4 and 8 weeks after surgery (*n* = 6). Results are expressed as the average value with corresponding standard deviation, and variability is indicated by standard deviation in the graphs. Group differences were analyzed using one‐way analysis of variance with subsequent Tukey post hoc testing. ns *p* > 0.05, ^*^ *p* < 0.05, ^**^ *p* < 0.01, ^***^ *p* < 0.001, ^****^ *p* < 0.0001.

Histological analysis was performed at two different time points after surgery. H&E staining revealed that the IVD structure in the ChS@HM group was preserved, displaying distinct boundaries between the NP and annulus fibrosus at two different time points postoperatively, and both the NP and annulus fibrosus structures remained maximally intact in the compression group. However, in the other treatment groups, the annulus fibrosus‐NP boundary was severely disrupted, and the annulus fibrosus also experienced structural damage. The structural integrity of the annulus fibrosus is essential for resisting the swelling pressure of the NP and maintaining uniform stress distribution. The relatively preserved lamellar structure of the annulus fibrosus in the ChS@HM group suggests that restoration of NP hydration may indirectly alleviate stress on the annulus fibrosus, thereby delaying its structural disruption and synergistically maintaining the overall mechanical stability of the IVD. Safranin O/fast green is used to evaluate glycosaminoglycan (GAG) content. The ChS@HM group exhibited higher collagen content compared to other compression groups at both weeks 4 and 8 (Figure 7A,B). According to the previously described method, histological scoring was performed based on the four categories of degenerative changes. Figure 7C,D shows the overall histological scores at two different time points postoperatively. The histological scores for ChS@HM were consistently significantly lower than those of the other treatment groups. These results indicate that ChS@HM has an NP regeneration effect in the body.

**FIGURE 8:**
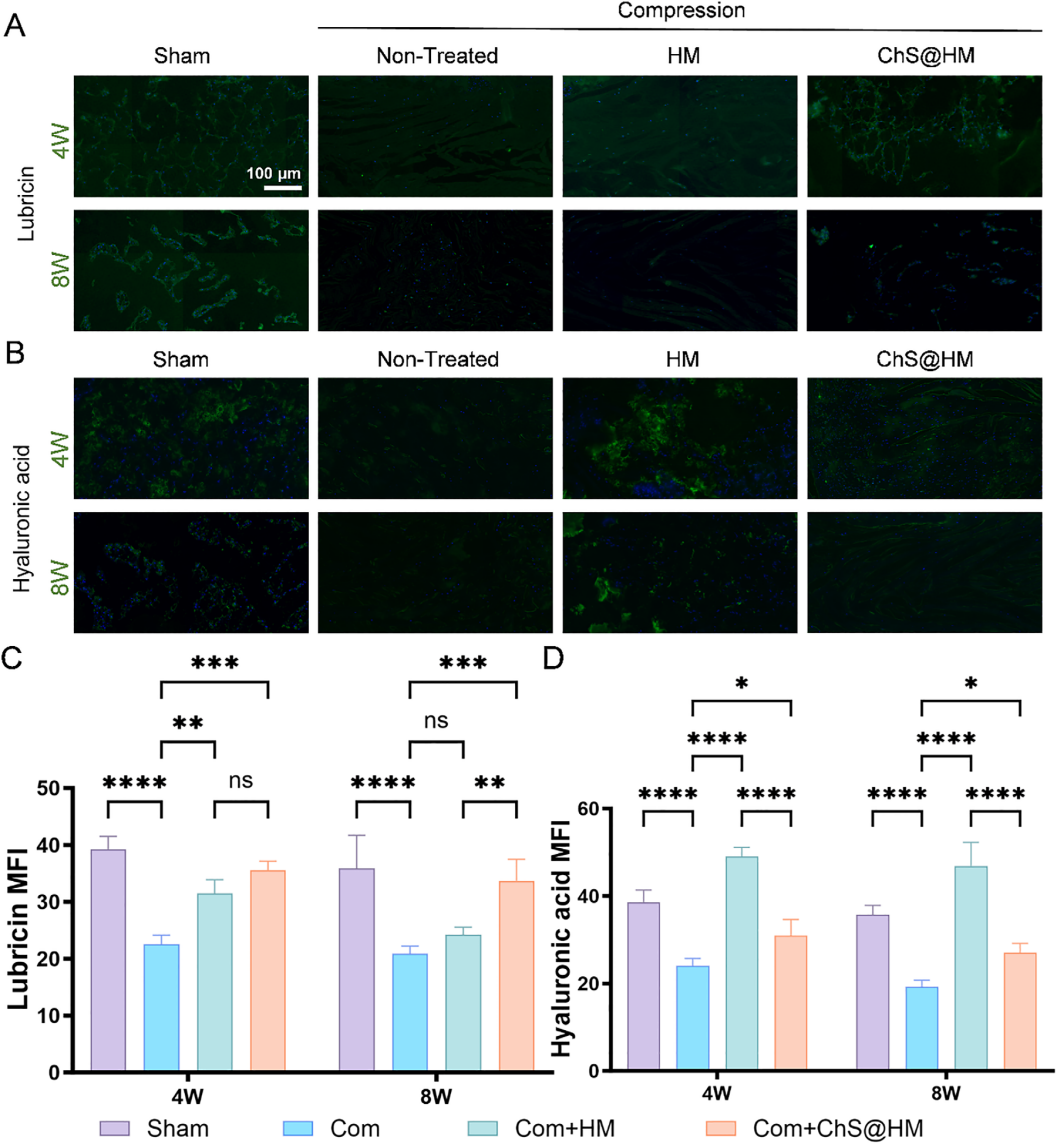
Immunofluorescence analysis of lubrication index in animal studies. (A) Fluorescent staining images of NP lubrication at 4 and 8 weeks after surgery. (B) Fluorescence images of hyaluronic acid staining in NP at 4 and 8 weeks after surgery. (C) Semi‐quantitative evaluation of NP lubricin fluorescence staining (*n* = 6). (D) Semi‐quantitative analysis of NP hyaluronic acid fluorescence staining (*n* = 6). Results are expressed as the average value with corresponding standard deviation, and variability is indicated by standard deviation in the graphs. Group differences were analyzed using one‐way analysis of variance with subsequent Tukey post hoc testing. ns *p* > 0.05, ^*^ *p* < 0.05, ^**^ *p* < 0.01, ^***^ *p* < 0.001, ^****^ *p* < 0.0001.

As a key boundary lubricant, lubricin (PRG4) reduces shear friction and mitigates local stress concentration by forming a low‐friction layer on tissue surfaces [32]. The immunofluorescence results showed significant differences in PRG4 among the groups. At 4 weeks, the sham group showed the strongest lubricin fluorescent signal, indicating that NP maintained good surface lubrication and cellular homeostasis. The compression group signal significantly decreased, indicating that mechanical stress inhibited the synthesis and secretion of PRG4. In contrast, the HM group partially restored the expression of lubricin, while the ChS@HM group showed a more significant rebound trend, indicating that the hydration network containing ChS helped enhance the stability of the lubricating layer and promoted the redistribution of PRG4. At week 8, the PRG4 signals in all groups generally decreased compared to those in week 4, but the overall trend remained consistent; for ChS@HM under compression conditions, a relatively high expression level of PRG4 could still be maintained. This suggests that the formation of a loose and more fluid hydration layer can sustainably improve the lubrication environment at the NP interface (Figure 8A,B). The immunofluorescence results of HA showed that the HM group exhibited the strongest fluorescence signal at 4 weeks, indicating that exogenous HA can significantly enhance hydration and hyaluronic acid accumulation in the NP matrix. The sham group maintained stable expression, while the compression group showed a significant decrease in signal, indicating that sustained mechanical loading inhibited HA synthesis. In contrast, although the ChS@HM group showed some recovery compared with the compression group, the signal was slightly lower than that of the HM group, mainly because of a decrease in the amount of HA provided in the formula. At 8 weeks, the overall signal of each group weakened, but the trend remained consistent, with the HM group still maintaining the highest level. Second, they were significantly higher than those of the compression group (Figure 8C,D). The hydration lubrication layer formed by HA facilitates sliding between collagen bundles and reduces collagen damage under mechanical loading. Therefore, the restoration of HA expression may contribute to maintaining collagen structural integrity, which is consistent with the better collagen preservation observed in the ChS@HM group in Figure 7. These results indicate that ChS@HM can synergistically maintain the hydration‐lubrication microenvironment and steady state of NP.

**FIGURE 9:**
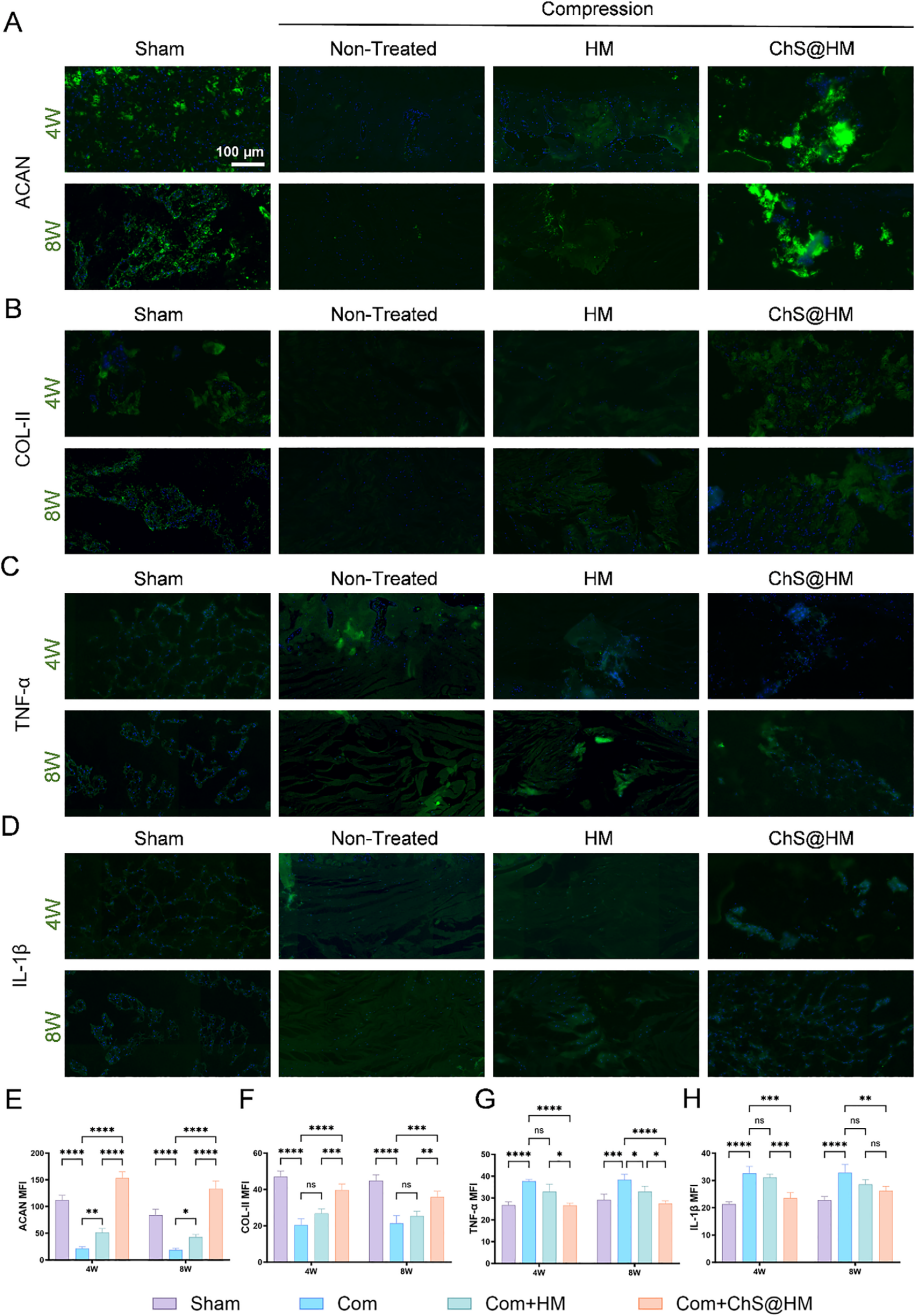
Immunofluorescence of synthetic catabolic metabolic indicators in animal experiments. (A) Fluorescence staining images of ACAN in NP at 4 and 8 weeks after surgery. (B) Postoperative fluorescence staining images of NP COL‐II at 4 and 8 weeks. (C) Fluorescence images showing TNF‐α staining in NP at two different time points. (D) Postoperative fluorescence staining images of IL‐1β in NP at two different time points. (E) Semi‐quantitative analysis of ACAN fluorescence staining of NP (*n* = 6). (F) Semi‐quantitative analysis of COL‐II fluorescence staining of NP (*n* = 6). (G) Semi‐quantitative analysis of TNF‐α fluorescence staining in NP (*n* = 6). (H) Semi‐quantitative analysis of IL‐1β fluorescence staining of NP (*n* = 6). Results are expressed as the average value with corresponding standard deviation, and variability is indicated by standard deviation in the graphs. Group differences were analyzed using one‐way analysis of variance with subsequent Tukey post hoc testing. ns *p* > 0.05, ^*^ *p* < 0.05, ^**^ *p* < 0.01, ^***^ *p* < 0.001, ^****^ *p* < 0.0001.

To further verify the reshaping effect of ChS@HM, NP metabolic homeostasis was evaluated by immunofluorescence staining to assess the level of NP synthesis metabolism. Aggrecan showed significant differences among the groups. After 4 weeks, the sham group maintained stable expression, while the compression group showed a significant decrease, indicating that sustained mechanical loading inhibited matrix protein synthesis. Partially restored expression was seen in the HM group. The strongest group signal of ChS@HM indicates that the introduction of exogenous ChS significantly promotes the accumulation of Aggrecan. At week 8, the signals of all groups decreased overall, but the trend remained consistent; the signals of the ChS@HM group continued to show significantly higher signals compared to the other compression groups (Figure 9A,E). In addition to providing glycosaminoglycan components as an exogenous source for the extracellular matrix, previous studies have shown that ChS also exerts anti‐inflammatory effects, reducing inflammation‐mediated Aggrecan degradation [33]. Moreover, as a natural glycosaminoglycan, it may promote cellular anabolism by mimicking the ECM microenvironment of the nucleus pulposus, thereby further enhancing matrix protein accumulation [34]. For COL‐II, the sham group maintained uniform expression at 4 weeks, while the compression group showed a significant decrease. The expression of the ChS@HM group significantly recovered at 8 weeks, although the signals in each group generally decreased. The ChS@HM group was still significantly higher than the other compression groups (Figure 9B,F). The results show that ChS@HM recovered the synthesis activity of Aggrecan and COL‐II in degenerated NP and reshaped the metabolic homeostasis of NP.

Abnormal mechanical force stimulation significantly increased TNF‐α and IL‐1β expression in NP, triggering an inflammatory cascade reaction and ultimately leading to impaired NP metabolic homeostasis [35]. The immunofluorescence results showed that at 4 weeks, the TNF‐α signals in all three compression groups were significantly enhanced, while the ChS@HM group could inhibit the inflammatory response to some extent. At 8 weeks, the overall inflammatory cytokine signals in each group increased, indicating a gradual accumulation of chronic inflammatory response induced by sustained mechanical load. The ChS@HM group still maintained relatively low TNF‐α expression (Figure 9C,G). For IL‐1β, the ChS@HM group showed similar inhibitory effects (Figure 9D,H). The above results indicate that ChS@HM can alleviate the chronic inflammatory response induced by mechanical stress by inhibiting the sustained upregulation of TNF‐α and IL‐1β, thereby promoting the reconstruction of NP metabolic homeostasis.

### 2.4 ChS@HM Analysis of Signal Pathways Regulating NP Metabolic Homeostasis

**FIGURE 10:**
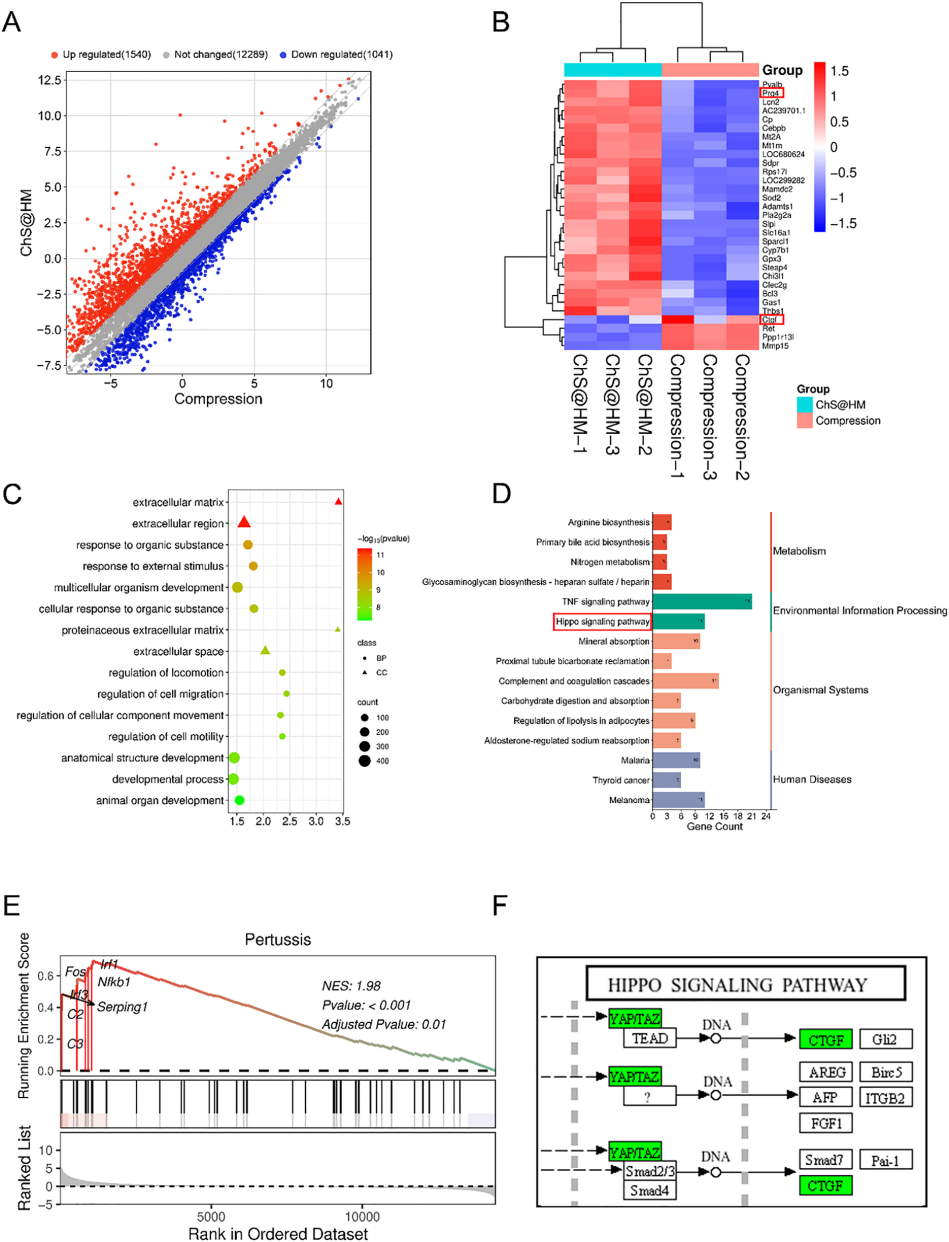
RNA sequencing analysis of Com and ChS@HM groups. (A) Scatter plot analysis of differentially expressed genes (DEGs) in Com and ChS@HM groups. (B) Heat map analysis of the Com and ChS@HM groups. (C) GO analysis on DEGs, and top‐15 significantly enriched entries. (D) The top 15 enriched pathways from the Kyoto Encyclopedia of Genes and Genomes (KEGG) in the Com and ChS@HM groups. (E) GSEA analysis shows that the Com and ChS@HM groups are associated with significant enrichment in the Pertusis pathway (*p* < 0.05). (F) Enrichment of DEGs in Hippo signaling pathway of Com and ChS@HM groups. Differential expression analysis was performed using standard RNA‐seq statistical pipelines with significance defined as *p* < 0.05.

To further analyze ChS@HM, we conducted RNA sequencing analysis on NP samples from rats at 4 weeks to investigate the regulatory mechanism of NP tissue in vivo. The analysis of differentially expressed genes (DEGs) showed that the ChS@HM group exhibited 676 upregulated and 428 downregulated genes (Figure 10A). Heatmap clustering displayed downregulation of the Ctgf gene in the ChS@HM group, suggesting that it may enhance overall stress dispersion and steady‐state maintenance ability by reducing local stress concentration and inhibiting YAP/CTGF axis activity. In addition, the Prg4, Adamts1, and inflammation regulatory gene Cp were related to NP hydration recovery and ECM remodeling. There was a significant upregulation in the ChS@HM group, indicating that this treatment regimen has a positive effect on the recovery of NP function (Figure 10B).

The GO functional annotation shows that DEGs are mainly enriched in entries such as the extracellular matrix, indicating that the ECM components of NP are regulated. The enrichment of the regulatory cell migration ratio developmental processes suggests that the tissue may undergo remodeling processes (Figure 10C). KEGG enrichment analysis further showed that DEGs significantly enriched the Hippo signaling pathway, suggesting that by regulating the local mechanical microenvironment to affect the Hippo/YAP mechanical transduction pathway, ChS@HM can promote ECM reconstruction. In addition, the enrichment of the TNF signaling pathway indicates that ChS@HM can regulate local inflammatory responses and synergistically maintain NP tissue homeostasis (Figure 10D). Next, in the Gene Set Enrichment Analysis (GSEA), the pertussis pathway was significantly enriched, which involves multiple inflammatory factors and receptors, further supporting the hypothesis that ChS@HM can promote the reconstruction of NP metabolic homeostasis by regulating local inflammatory signals (Figure 10E). Based on the above results, combined with the downregulation of Ctgf and enrichment of the Hippo pathway, ChS@HM may be regulated through the Hippo/YAP pathway mediated by stress dispersion, inhibiting the fibrosis process mediated by CTGF and promoting the ECM steady‐state reconstruction of NP (Figure 10F).

To corroborate the RNA sequencing findings at the protein level, NP tissues were subjected to Western blot analysis. In comparison with the control groups, CTGF protein levels were markedly suppressed following ChS@HM treatment, whereas a clear elevation in phosphorylated YAP was observed, while total YAP remained largely unchanged. These protein‐level alterations are consistent with a modulation of Hippo signaling activity and suggest that the therapeutic effect of ChS@HM is associated with attenuation of CTGF‐related fibrotic responses, ultimately contributing to the re‐establishment of extracellular matrix homeostasis within the NP (Figure S7).

## 3 Conclusion

In this study, we successfully constructed a millimeter‐scale structure based on microfluidic and photo‐cross‐linking technology. Biomimetic ChS@HM hydrated millimeter spheres, which simulate the structural characteristics of natural NP, rely on the covalent cross‐linking network of ChSMA and HAMA to achieve a breakthrough in structural biomimetics and functional synergy. In vitro and in vivo experiments verified that this millimeter sphere effectively overcomes the limitations of traditional microspheres, such as stress concentration and difficulty in maintaining long‐term mechanical properties owing to their small size, significantly improving the stress dispersion ability of tissues. Simultaneously, the anti‐inflammatory properties of ChS synergistically alleviate local inflammatory responses, promote the repair of the NP metabolic microenvironment, and restore hydration status. Overall, the hydrated millimeter sphere exhibits good biocompatibility and multiple functions, providing innovative material strategies and a theoretical basis for the regulation of inflammatory microenvironment and tissue regeneration related to IDD.

## 4 Experimental Section

Complete details of materials, reagents, and experimental procedures are provided in the Supporting Information. Key methods relevant to the main figures are described below.

### 4.1 Preparation and Characterization of HAMA and ChSMA

Hyaluronic acid (MW 34.5 kDa, 2 wt% in deionized water, pH 8.0) was reacted with methacrylic anhydride under stirring in an ice bath for 24 h. The reaction mixture was purified by dialysis in deionized water for 72 h, followed by freeze‐drying, yielding HAMA as a white powder. The same procedure was applied to ChS to obtain ChSMA. The methacrylation degrees were determined by ^1^H NMR (400 MHz, Bruker) based on the integral ratio of methacryloyl protons to N‐acetyl methyl protons.

### 4.2 Preparation and Characterization of Hydrogel Millimeter Spheres

According to the proportion of HA and ChS in NP [24], HAMA (0.4 wt%) and ChSMA (1.6 wt%) were mixed with photoinitiator to form a precursor solution. The solution was introduced into a microfluidic flow‐focusing device to generate uniform droplets in an oil phase, followed by ultraviolet‐induced crosslinking to obtain hydrogel millimeter spheres (HM and ChS@HM). The resulting spheres were washed and stored at 4°C prior to further use. Surface morphology and internal microstructure were characterized by scanning electron microscopy.

### 4.3 Degradation Test of Hydrogel Millimeter Spheres

ChS@HM spheres were incubated in hyaluronidase solution (1000 U/mL) at 37°C with shaking. A fresh enzyme solution was supplied every 48 h. Mass loss was monitored at specified time points, and the remaining mass percentage was calculated relative to the initial weight.

### 4.4 Finite Element Simulation

Solid mechanics simulations were performed using COMSOL Multiphysics (v6.1). The model comprised a cylinder (radius 510 µm, height 1100 µm) containing either one millimeter sphere (1 mm diameter) or 480 microspheres (100 µm diameter). Hydrogel was modeled as a linear elastic material, and the cylinder as a high‑strength alloy. A fixed constraint was applied on the bottom surface, and a point load of 4.5 N was applied on the top surface. The mesh was refined around the spheres. Stress distribution and load dispersion were analyzed.

### 4.5 Lubrication Test of Hydrogel Millimeter Spheres

A universal materials tester (SRV5) was used to conduct tribological tests in linear reciprocating mode. The upper friction pair was a polytetrafluoroethylene plate, and the lower pair was a GCr15 steel ball. HM spheres, ChS@HM spheres, or ChS solution (10 mg/mL, 15 mL) were used as lubricants. Tests were conducted at normal loads of 1 N, 5 N, and 10 N, with an amplitude of 4 mm, frequency of 1 Hz, and duration of 600 s.

### 4.6 Preparation of Extract Liquids

The procedure was adapted from a published method with slight adjustments [36]. Each type of sphere was incubated in serum‐free medium at a concentration of 0.1 g/mL at 4°C for 48 h. After incubation, the supernatant was harvested and passed through a filter to obtain the corresponding extracts.

### 4.7 Establishment of a Static Compression Model for IDD in Rats

All animal experiments adhere to the guidelines approved by the Animal Ethics Committee of Yangpu Hospital, affiliated with Tongji University (LL‐2025‐SCI‐004). Ten‐week‐old SD rats were obtained from a commercial supplier and acclimated for one week. As mentioned earlier, a mechanical IDD model was established by applying a constant static compression force of 4.5 N to the C8/9 and C9/10 segments of the rat caudal vertebrae using a compression device [37]. Subsequently, PBS and millimeter spheres were injected into the intervertebral space. The compression device was removed 2 weeks after surgery.

### 4.8 Animal Imaging Evaluation

It involved recording X‐ray and MRI images of rat tail vertebrae at 4‐ and 8‐week post‐surgery. The IVD was assessed with X‐rays and analyzed using ImageJ, with DHI% calculated as per established methods. A T2‐weighted MRI scan of the caudal region was performed to assess IVD water content. The average grayscale value was measured using ImageJ software.

### 4.9 Statistical Analysis

The normality of the data distribution was evaluated using the Shapiro–Wilk test, and no significant deviations from normal distribution were detected. Results are presented as mean ± standard deviation (SD). Each in vitro assay was conducted using three separate biological samples (*n* = 3), while in vivo studies included six animals per group (*n* = 6). For datasets involving more than two groups, statistical significance was determined by applying a one‐factor variance analysis (ANOVA), with subsequent post hoc pairwise evaluation to resolve intergroup differences. In cases limited to two‐group comparisons, an independent sample t‐test was employed using a two‐sided testing strategy. A *p* value < 0.05 was considered statistically significant. GraphPad Prism (version 8.0) was used as the platform for all data processing and statistical evaluation.

## Conflicts of Interest

The authors declare no conflict of interest.

## Supporting information




**Supporting file 1**: advs75249‐sup‐0001‐SuppMat.docx

## Data Availability

The data that support the findings of this study are available from the corresponding author upon reasonable request.
