# Supplementary material for: In Situ Stress‐Dispersing Hydrogel Millispheres via Load Redistribution to Restore Nucleus Pulposus Metabolic Homeostasis
Source: Adv Sci (Weinh). 2026 Apr 13:e75249. Online ahead of print. doi: 10.1002/advs.75249 (PMC13335095; doi:10.1002/advs.75249)
Supplement: Supplementary file 1 — Supporting file 1: advs75249‐sup‐0001‐SuppMat.docx [file ADVS-9999-e75249-s001.docx]

Supporting Information

**In Situ Stress-Dispersing Hydrogel Millispheres via Load Redistribution to Restore Nucleus Pulposus Metabolic Homeostasis**

*Ang Li,* *Hui Yuan, Honglei Xiao, Xiaodong Liu*, Wenguo Cui**

A. Li, H. Xiao, X. Liu

Department of Orthopedics, Yangpu Hospital, School of Medicine, Tongji University, Shanghai 200090, China.

Center for Clinical Research and Translational Medicine, Yangpu Hospital, School of Medicine, Tongji University, Shanghai 200090, China.

E-mail addresses: xiaodong.liu@tongji.edu.cn (Xiaodong Liu)

H. Yuan, Prof. W. Cui

Department of Orthopaedics, Shanghai Key Laboratory for Prevention and Treatment of Bone and Joint Diseases, Shanghai Institute of Traumatology and Orthopaedics, Ruijin Hospital, Shanghai Jiao Tong University School of Medicine, 197 Ruijin 2nd Road, Shanghai 200025, P. R. China.

E-mail: wgcui80@hotmail.com (Wenguo Cui)

**Table S1.** Primers used for qRT-PCR.

| Primer | Orientation | Sequence (5´-3´) |
| --- | --- | --- |
| TNF-α | Forward | ACTGAACTTCGGGGTGATCG |
|  | Reverse | GCTTGGTGGTGTGTGTACGAC |
| IL-1β | Forward | GACTTCACCATGGAACCCGT |
|  | Reverse | GGAGACTGCCCATTCTCGAC |
| ACAN | Forward | GACCTGTGTGAGATCGACCA |
|  | Reverse | GTTGGTTTGGACGCCACTTC |
| COL-II | Forward | GGGAATGTCCTCTGCGATGAC |
|  | Reverse | GAAGGGGATCTCGGGGTTG |
| MMP13 | Forward | CAAGCAGCTCCAAAGGCTAC |
|  | Reverse | TGGCTTTTGCCAGTGTAGGT |
| GAPDH | Forward | GACAGCCGCATCTTCTTGTG |
|  | Reverse | ATCCGTTCACACCGACCTTC |


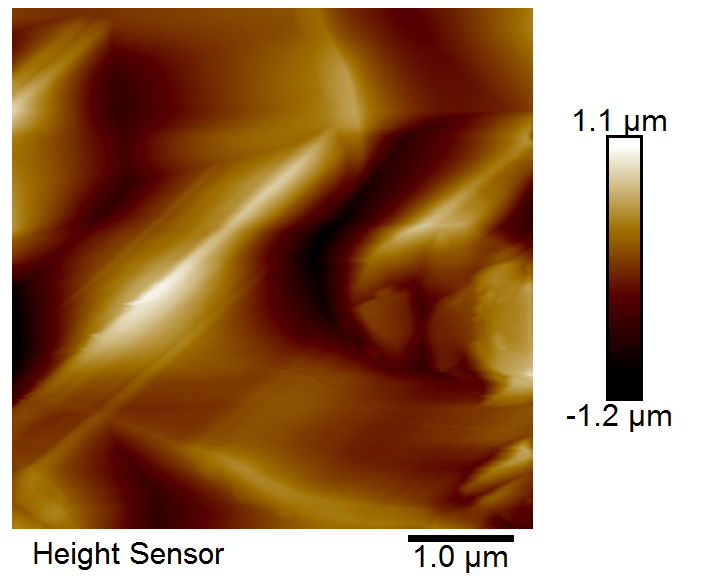

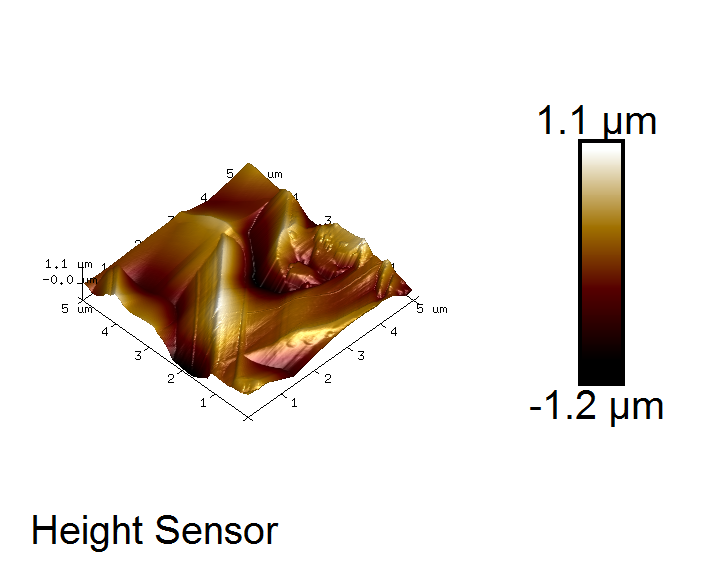
**Figure**

**Figure S1.** **AFM images of ChS@HM millimeter spheres.**


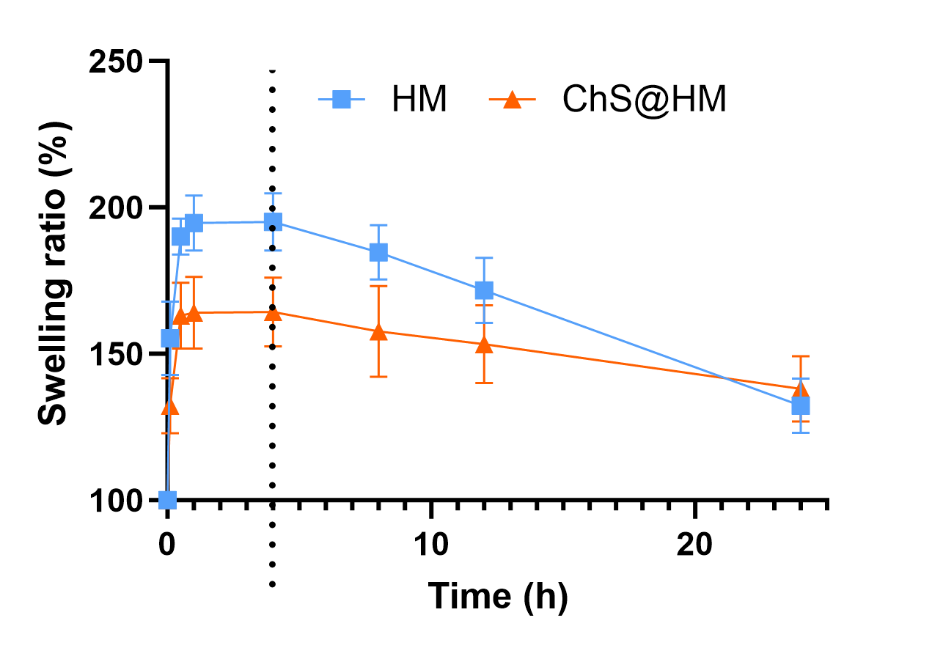


**Figure S2.** **Swelling and water retention properties of HM and ChS@HM millimeter spheres.**


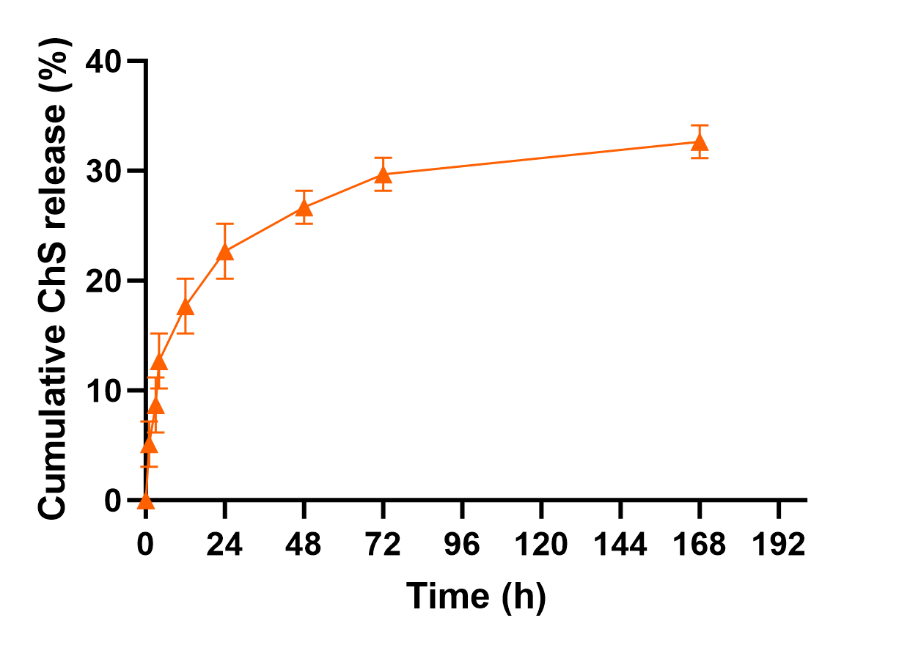


**Figure S3.** **In vitro release profile of ChS from ChS@HM millimeter spheres.**


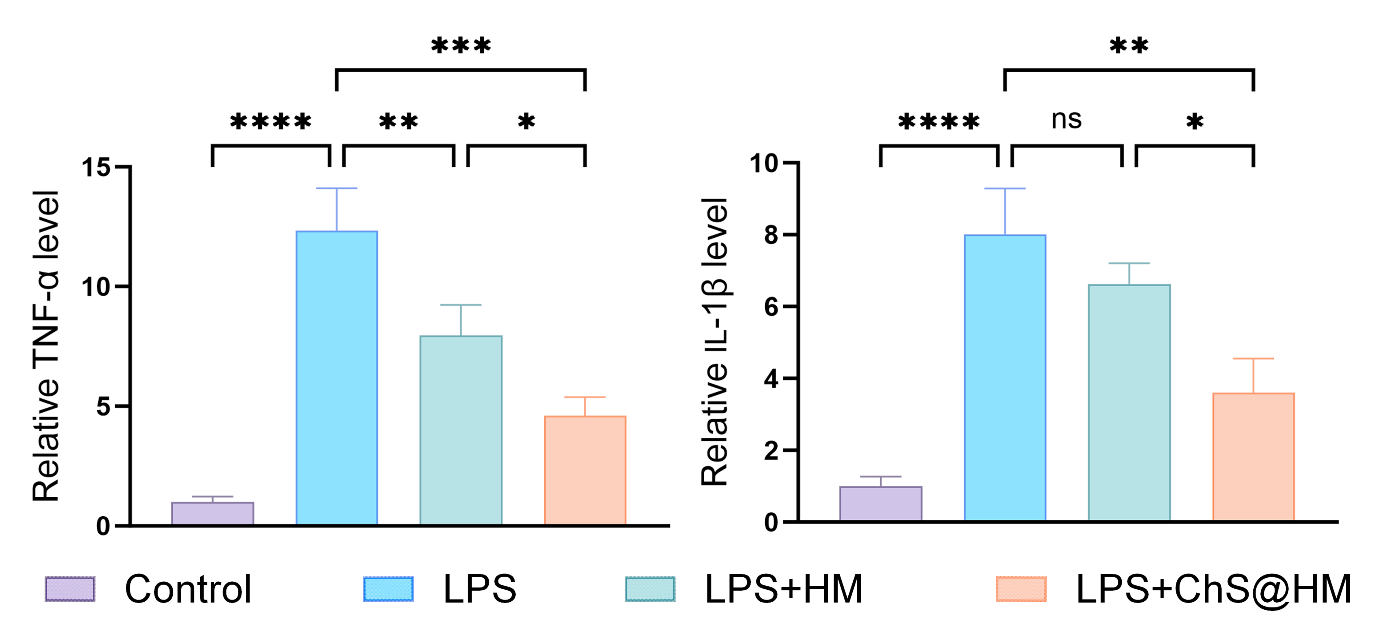


**Figure S4.** **Relative mRNA expression levels of TNF-α and IL-1β.**


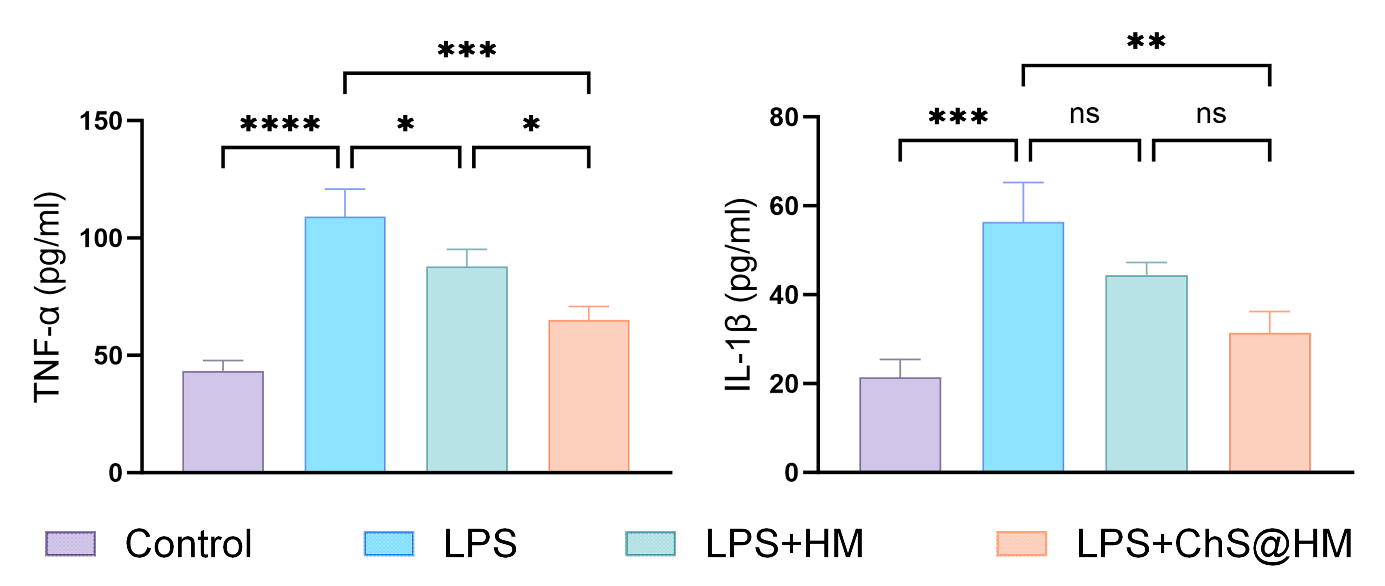
**Figure S5.** **ELISA detection of the concentrations of inflammatory cytokines TNF-α and IL-1β in cell supernatant.**


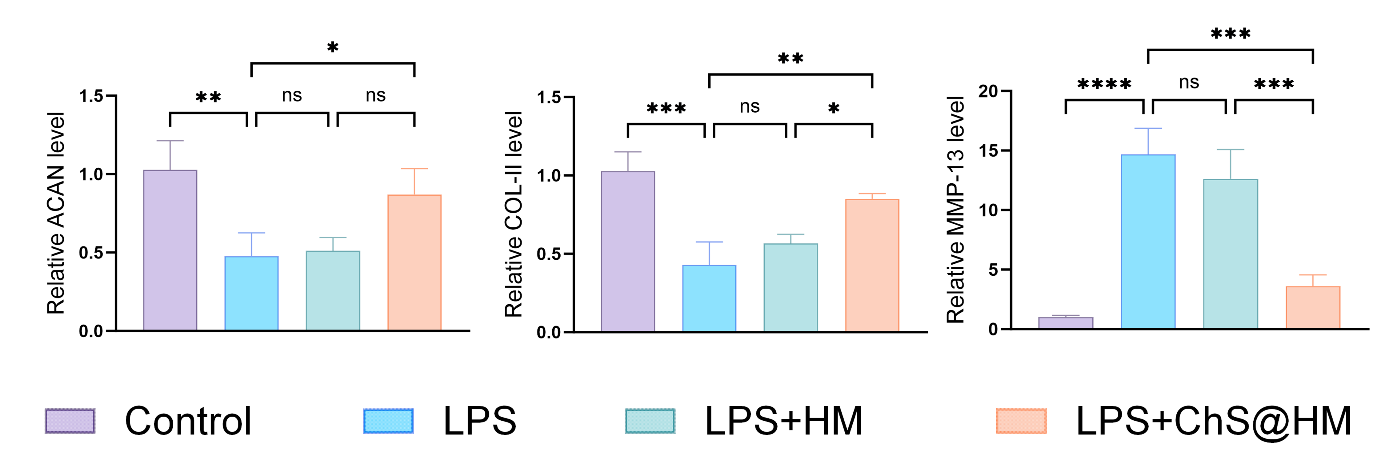
**Figure S6.** **Relative mRNA expression levels of Aggrecan, COL-II and MMP-13.**


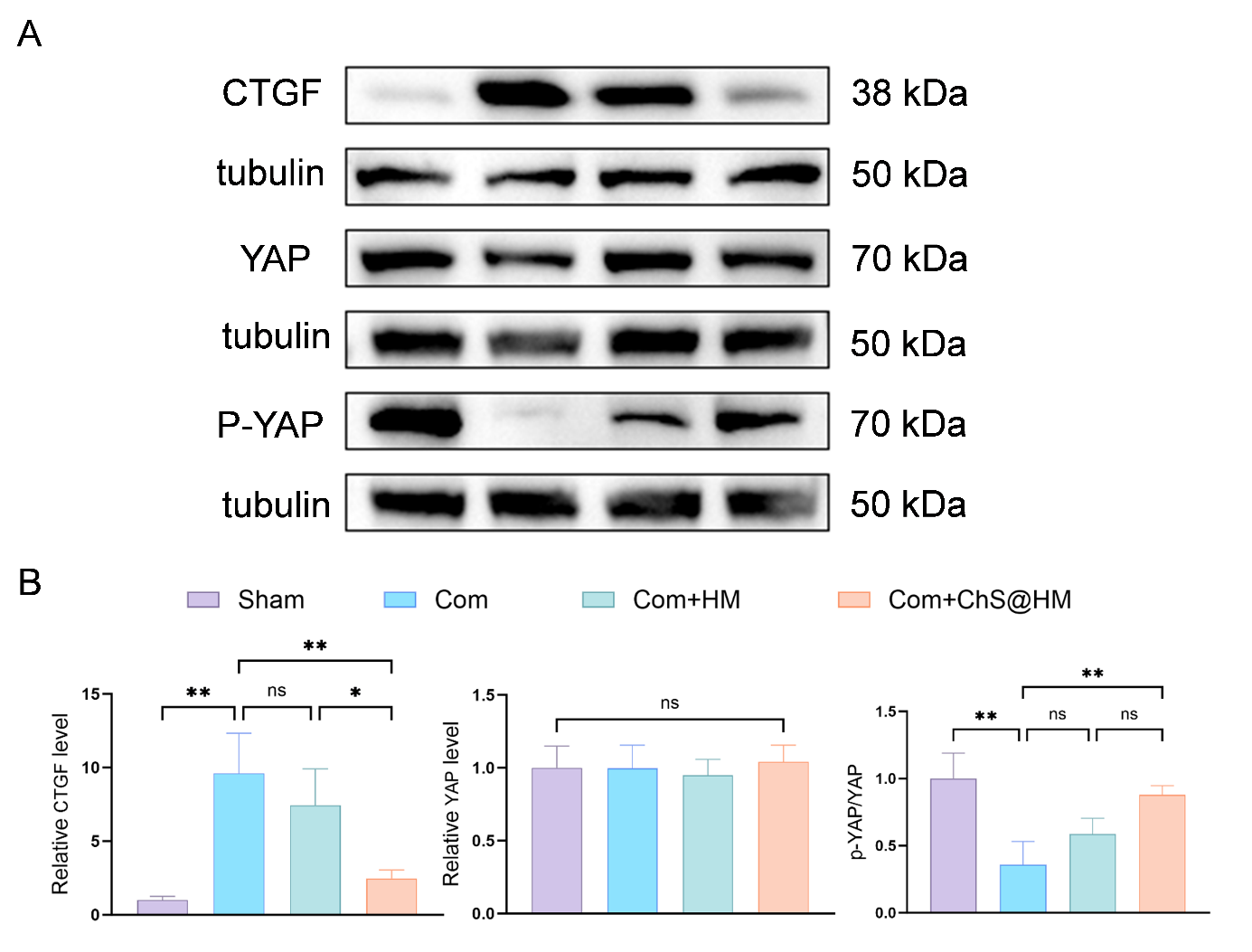
**Figure S7.** **Western blot analysis was used to estimate the pathway protein levels in rat nucleus pulposus tissues.** **A) Representative Western blot bands of CTGF, YAP, p-YAP, and Tubulin. B) Semiquantitative analysis of CTGF expression normalized to Tubulin. C) Semiquantitative analysis of total YAP expression normalized to Tubulin. D) Semiquantitative analysis of the p-YAP/YAP ratio, expressed as fold change relative to the control group.**


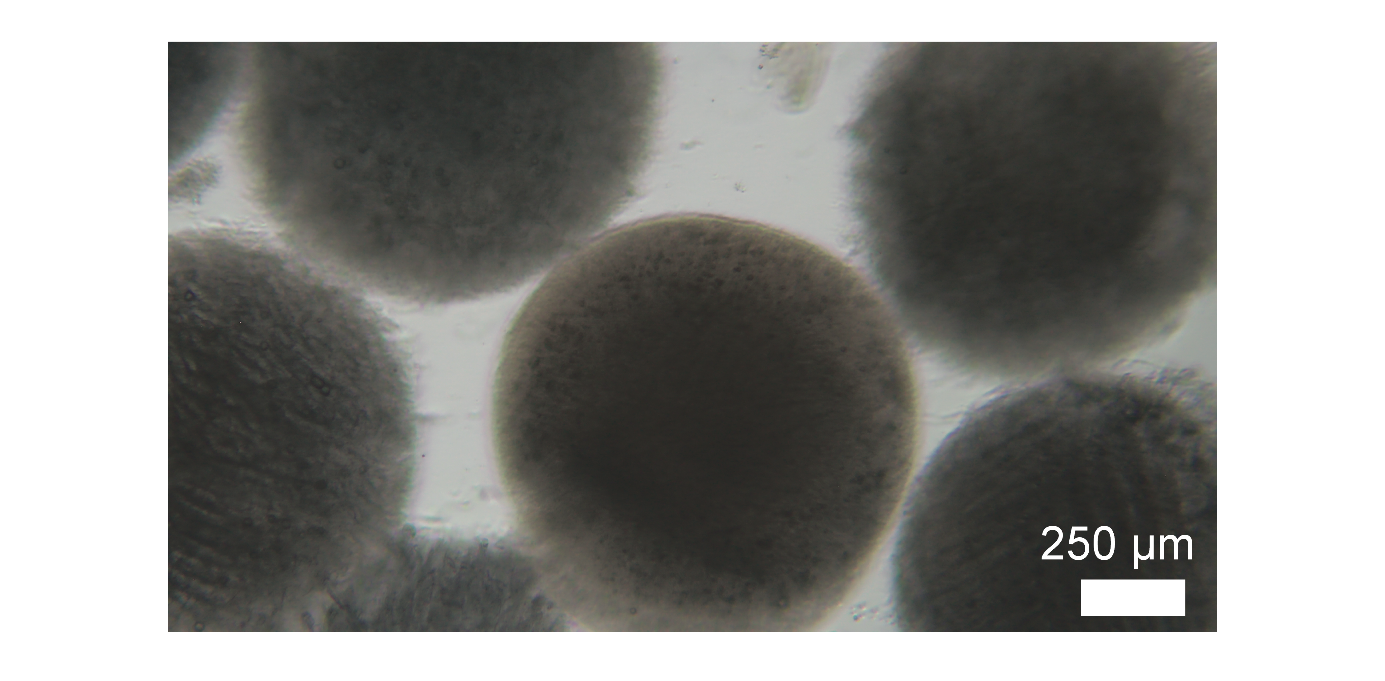
**Figure S8.** **Bright-field image of ChS@HM millispheres.**

**Supplementary** **Methods**

*Swelling and water retention experiment*: The swelling behavior of hydrogel spheres was evaluated in PBS (37 °C) by measuring mass change over time until equilibrium was reached. Water retention was assessed by monitoring mass loss during dehydration under ambient conditions after full swelling.

*Release curve experiment*: Soak the ChS@HM millimeter spheres in PBS (pH 7.4, 37°C) and incubate them on a shaker (80 rpm). Collect the supernatant at preset time points and replenish with an equal volume of fresh PBS. Measure the ChS content in the supernatant using the dimethyl methylene blue method, draw a standard curve using chondroitin sulfate as the standard, and calculate the cumulative release percentage.

*Biocompatibility Evaluation:* Sterilized ChS@HAMA millimeter spheres were placed in the upper compartment of a 48 well plate, while NP cells were placed in the lower chamber. On days 1, 3, and 5, cells were incubated with the calcein AM/PI cell viability/cytotoxicity assay kit for 30 min and observed under a fluorescence microscope. In addition, changes in cell proliferation ability were evaluated using a CCK8 assay kit.

*Immunofluorescence Staining*: LPS was added to different groups of extracts to a final concentration of 100 μg/mL and cocultured with NP cells. Normal cultured cells were used as the negative control group, while cells containing only LPS but no extract were used as the positive control group. After coculturing for 12 h, the cells underwent three PBS washes and were subsequently fixed in 4% paraformaldehyde for 30 minutes. Subsequently, Cells were exposed to 0.1% Triton X-100 for 1 minute to increase cell permeability. Next, the cells were incubated overnight at 4°C with primary antibodies against TNF-α, IL-1β, ACAN, COL-II, and MMP13. The next day, cells were incubated with the corresponding secondary antibodies for 1 hour at room temperature. After washing thrice with PBS, the cells were counterstained with DAPI and visualized using a fluorescence microscope. The fluorescence signal intensity was quantitatively analyzed using ImageJ.

*Inflammatory Cytokine Detection*: After treating the cells using the same procedure as described above, the supernatant was collected and centrifuged to eliminate cell debris. An ELISA assay kit was used to detect changes in inflammatory factors. The sample was added to the plate at 37°C for 90 min, waste liquid was discarded, and biotinylated antibody working solution was added at 37°C for 60 min. After discarding the waste liquid, the plate was rinsed three times. HRP conjugate working solution was then added and incubated at 37°C for 30 minutes. Following this, the waste liquid was discarded again, TMB was added, and the plate was incubated at 37°C for 15 minutes. The absorbance value was recorded at 450 nm wavelength after adding the stop solution.

*Histological Evaluation:* Following in vivo imaging assessment, the rats were euthanized, and IVD specimens were collected for histological evaluation. Samples were fixed in 4% paraformaldehyde for 24 hours, decalcified in EDTA for 4 weeks, embedded in paraffin, and sectioned at 5 μm for H&E and safranin O/fast green staining. Cells were observed under a light microscope and histologically classified.

*Immunofluorescence Evaluation of Animal Slices:* Immunofluorescence assay was conducted on animal slices to assess the expression levels of lubricin, hyaluronic acid, ACAN, COL-II, TNF-α, and IL-1β in IVDs. Tissue sections were treated with 0.1% Triton X-100 for 15 minutes to permeabilize them, followed by a 30-minute blocking step using goat serum. Primary antibodies targeting ACAN, COL II, TNF-α, and IL-1β were incubated overnight at 4℃. Sections were incubated with suitable secondary antibodies for 2 hours at room temperature, followed by DAPI staining of cell nuclei. Fluorescence images were captured with a fluorescence microscope.

*RNA Sequencing and Western Blot:* Rat NP samples were assigned to compression and compression plus treatment groups and harvested at 4 weeks, with six replicates per group. Tissue samples were rapidly frozen in liquid nitrogen and stored at −80 °C prior to RNA extraction. For Western blot, total protein was extracted from rat nucleus pulposus tissue by RIPA lysis buffer homogenate, and protein concentration was determined by BCA method. The equal amount of protein was separated by 10% SDS-PAGE and transferred to a PVDF membrane. After being sealed with 5% skim milk for 1 hour, the primary antibodies YAP, p-YAP, CTGF, and Tubulin were incubated overnight at 4° C. The next day, HRP labeled secondary antibody was incubated at room temperature for 1 hour and ECL chemiluminescence was developed. Quantitative analysis of band grayscale values using ImageJ software, with Tubulin as an internal reference.
